# Therapeutic Potential of Injectable Nano-Mupirocin Liposomes for Infections Involving Multidrug-Resistant Bacteria

**DOI:** 10.3390/pharmaceutics13122186

**Published:** 2021-12-17

**Authors:** Ahuva Cern, Yaelle Bavli, Atara Hod, Daniel Zilbersheid, Shazad Mushtaq, Ayelet Michael-Gayego, Dinorah Barasch, Yael Feinstein Rotkopf, Allon E. Moses, David M. Livermore, Yechezkel Barenholz

**Affiliations:** 1 Laboratory of Membrane and Liposome Research, Department of Biochemistry, The Hebrew University of Jerusalem, Jerusalem 9112102, Israel; ahuva.cern@mail.huji.ac.il (A.C.); yaellef@ekmd.huji.ac.il (Y.B.); atara87@gmail.com (A.H.); danielzilb@savion.huji.ac.il (D.Z.); 2 Antimicrobial Resistance and Healthcare-Associated Infections Reference Unit, UK Health Security Agency, London NW9 5HT, UK; SHAZAD.MUSHTAQ@phe.gov.uk; 3 Department of Clinical Microbiology & Infectious Diseases, Hadassah Hebrew University Medical Center, Jerusalem 9112102, Israel; ayeletg@hadassah.org.il (A.M.-G.); mosesallon@gmail.com (A.E.M.); 4 The Mass Spectrometry Unit, School of Pharmacy, The Hebrew University of Jerusalem, Jerusalem 9112102, Israel; dinorah.barasch@mail.huji.ac.il; 5 Light Microscopy Laboratory, Core Research Facility, Faculty of Medicine, The Hebrew University of Jerusalem, Jerusalem 9112102, Israel; yaelfe@savion.huji.ac.il; 6 Norwich Medical School, University of East Anglia, Norwich NR4 7TJ, UK; livermor@claranet.co.uk

**Keywords:** nano-liposomes, mupirocin, multi-drug resistant bacteria, injection, pharmacokinetics, cross resistance, *Neisseria gonorrhoeae*, vaginal distribution, vancomycin-resistant *E. faecium*

## Abstract

Antibiotic resistance is a global health threat. There are a few antibiotics under development, and even fewer with new modes of action and no cross-resistance to established antibiotics. Accordingly, reformulation of old antibiotics to overcome resistance is attractive. Nano-mupirocin is a PEGylated nano-liposomal formulation of mupirocin, potentially enabling parenteral use in deep infections, as previously demonstrated in several animal models. Here, we describe extensive in vitro profiling of mupirocin and Nano-mupirocin and correlate the resulting MIC data with the pharmacokinetic profiles seen for Nano-mupirocin in a rat model. Nano-mupirocin showed no cross-resistance with other antibiotics and retained full activity against vancomycin-, daptomycin-, linezolid- and methicillin- resistant *Staphylococcus aureus*, against vancomycin-resistant *Enterococcus faecium*, and cephalosporin-resistant *Neisseria gonorrhoeae*. Following Nano-mupirocin injection to rats, plasma levels greatly exceeded relevant MICs for >24 h, and a biodistribution study in mice showed that mupirocin concentrations in vaginal secretions greatly exceeded the MIC_90_ for *N. gonorrhoeae* (0.03 µg/mL) for >24 h. In summary, Nano-mupirocin has excellent potential for treatment of several infection types involving multiresistant bacteria. It has the concomitant benefits from utilizing an established antibiotic and liposomes of the same size and lipid composition as Doxil^®^, an anticancer drug product now used for the treatment of over 700,000 patients globally.

## 1. Introduction

Nano-mupirocin is a formulation of PEGylated nano-liposomes loaded with mupirocin, an antibiotic with a unique mode of action and no cross-resistance. The specific target is the isoleucine-binding site on the bacterial isoleucyl-transfer-RNA synthetase, as demonstrated for *Staphylococcus aureus* and *Escherichia coli*—though the latter species is inherently resistant owing to impermeability [1]. Mupirocin was approved by the FDA in 1997 but is limited to topical use owing to rapid systemic elimination and high protein binding [2,3,4].

Computational machine-learning identified mupirocin as a highly suitable candidate for nano-liposomal delivery [5,6,7]. Remote active loading protects circulating drug from metabolism and promotes accumulation at an infection site, facilitated by leaky vessels and low lymphatic clearance [8]. Nano-mupirocin’s activity after injection has been confirmed in mouse models of necrotizing fasciitis, osteomyelitis and pneumonia, also rabbit endocarditis [9,10]. A mouse study showed higher plasma levels and a much longer half-life than for the free drug (4.4 h vs. 5 min), with this pharmacokinetic pattern confirmed in rabbits [9]. In addition, we showed that mupirocin retained antibacterial activity despite being encapsulated in the intraliposomal aqueous phase. Nano-mupirocin is taken-up by macrophages, killing internalized bacteria [10]. The antibacterial inactivity of the unloaded liposomes was previously demonstrated both in vitro and in vivo [10].

Here, we further describe in vitro profiling of nano-mupirocin, including its effect on resistant strains and in relation to pharmacokinetic (PK) and biodistribution (BD) studies.

## 2. Materials and Methods

### 2.1. Materials

Mupirocin was received from Teva (Debrecen, Hungary); hydroxy-propyl β-cyclodextrin (HPCD) from Roquette Frères (Lestrem, France); hydrogenated soy phosphatidylcholine (HSPC), 1,2-distearoyl-sn-glycero-3-phosphoethanolamine-N-[methoxy(polyethylene glycol)-2000 Da] (mPEG DSPE) and cholesterol from Lipoid GmbH (Ludwigshafen, Germany); Sepharose CL-4B from GE Healthcare (Little Chalfont, UK); mycophenolic acid from Sigma-Aldrich (Saint Louis, MO, USA); adult bovine serum from Biological Industries (Beit Haemek, Israel); LC/MS-grade acetonitrile (ACN), methanol (MeOH) and water from Biolab Ltd. (Jerusalem, Israel); formic acid (FA) from J.T. Baker (Phillipsburg, NJ, USA). Analytical solvents were HPLC grade; other chemicals were commercial reagent grade.

Tryptic Soy Agar (TSA) plates containing 5% sheep blood and Chocolate Agar plates were from Liofilchem (Roseto degli Abruzzi, Italy). Cation-adjusted Mueller-Hinton broth (CA-MHB) and GC medium base were from Becton Dickinson (Franklin Lakes, NJ, USA). CA-MHB with TES (2-[[1,3-dihydroxy-2-(hydroxymethyl)propan-2-yl]amino]ethanesulfonic acid) was from TREK Diagnostic Systems (East Grinstead, UK).

### 2.2. Methods

#### 2.2.1. Nano-Mupirocin Production

Nano-mupirocin was prepared as described previously [9,10]; brief details are given in the Supplementary Information. The nanoliposome size was 74 nm with a polydispersity index (PDI) of 0.05. Values of D10, D50 and D90 were 54, 76 and 108 nm, respectively, with a SPAN of 0.71. The total mupirocin concentration in the formulation was 6.56 mg/mL, of which 5.65 mg/mL was liposome encapsulated (86% loading). The intraliposomal (trapped) volume was calculated as 5.94% of total volume, and 0.13% trapped volume per mg lipids. The trapped volume calculation was based on determination of calcium concentrations, as these reflect the volume of the hydration medium trapped in the liposomes. The pH of the dispersion was 6.3, whereas the intra-liposomal pH was 8.4 before remote active loading of mupirocin and 7.7 after mupirocin loading (10). Nano-mupirocin liposomes fluorescently labeled with lissamine-rhodamine B phosphatidylethanolamine (LRPE) were prepared as previously described [10].

Nano-mupirocin is a liquid dispersion and was used ‘as is’ or diluted in the desired aqueous solution when required.

#### 2.2.2. Quantification of Mupirocin 

Mupirocin was assayed by HPLC, using isocratic elution with a 75:25 (*v*/*v*) mobile phase of 50 mM sodium phosphate pH 6.3: acetonitrile at a flow rate of 1.0 mL/min on a Luna C18 column, 5 μm, 4.6 mm × 150 mm (Phenomenex, Torrance, CA, USA). The injection volume was 20 μL and detection was by UV absorption at 229 nm. Samples for determination of total mupirocin were diluted in methanol. Free (non-liposomal) mupirocin was determined after ultrafiltration on an Amicon Ultra 100K device (Millipore Corp). Levels of liposomal mupirocin were calculated by subtracting free from total mupirocin after correction for non-specific drug adsorption by the filter. Percent free drug was calculated by dividing free mupirocin by the total mupirocin concentration in the formulation.

#### 2.2.3. Antimicrobial Susceptibility Testing

MICs were measured by CLSI broth microdilution [11,12] with heavier inocula additionally used in some experiments. Minimal bactericidal concentrations (MBCs) were determined according to the CLSI guideline M26-A [13], with the MBC defined as the lowest drug concentration to kill 99.9% of the test inoculum. These in vitro studies were performed at International Health Management Associates (IHMA Europe. Monthey, Switzerland) and Public Health England (London, UK).

#### 2.2.4. Resistance Selection

##### Single-Step Antimicrobial Resistance Selection

Bacterial suspensions were adjusted to a 4 McFarland and concentrated 10-fold by centrifugation in 0.9% NaCl, with 100-μL volumes (c. 10^9^ CFU) then spread onto Tryptic Soy Agar (TSA) containing 5% sheep blood (Liofilchem) and mupirocin or Nano-mupirocin at 4, 8, or 16 × MIC, as determined with inocula of 10^10^ CFU/mL. Free mupirocin was added from a solution prepared in DMSO and diluted 100-fold in molten Mueller-Hinton agar (MHA), then poured in Petri dishes. Nano-mupirocin was diluted at 100 × the test concentration required, with 200 μL volumes of these solutions spread on plates containing 19.8 mL of solidified MHA. These were dried at room temperature before inoculation. This method was used to prevent the degradation of Nano-mupirocin liposomes, which occurs at temperatures above 40 °C, as needed to keep agar molten. Colonies growing after incubation for 24 h were enumerated relative to those that grew when dilutions of the same inoculum were plated onto drug-free blood-supplemented TSA. Two mutants per series had MICs determined after 5 sub-cultures on drug-free agar. These studies were performed at IHMA.

##### Multi-Step Antimicrobial Resistance Selection

Bacteria were grown in broth containing antibiotic at 0.5 × MIC, with this growth then used to inoculate a dilution series. Samples from the highest concentration allowing growth was then used, on the next day, to inoculate a further dilution series, with this process repeated for 15 days. Isolates from cultures where the MIC increased > 2-fold were stored frozen, as were those from all Day 15 cultures. Stored isolates were passaged five times on antibiotic-free media, with MICs then re-determined to test whether stable resistance had been selected. These experiments were performed by IHMA.

#### 2.2.5. Nano-Mupirocin Pharmacokinetics (PK) in Rats

As part of a wider toxicology study, performed at ITR Laboratories (Baie-d'Urfé, QC,Canada) and not presented in detail here, Nano-mupirocin was administered to Sprague Dawley Crl:CD (SD) rats aged 7–8 weeks (Charles River Canada Inc., Saint-Constant, QC, Canada) on Days 1, 4, 7, 9, 11 and 14. Test groups comprised 9 males and 9 females; the control group comprised 3 males and 3 females. Dosing groups received different volumes of the same formulation: IV groups thereby received 10, 30 and 100 mg/kg by bolus over >2 min into the tail vein; Intra-muscular (IM) group received 0.2 mL at each of two sites, totaling 2.6 mg/animal (10.5 mg/kg, assuming a 250 g body weight); control animals received 15.24 mL/kg vehicle. The doses were calculated based on the total mupirocin concentrations in the formulation.

Blood samples were collected from 3 animals/sex/time point by jugular venipuncture into K_2_EDTA tubes. Following its last sampling, each animal was euthanized. Sampling timepoints on Days 1 and 14, were 5 min, 0.5, 1, 2, 4, 8, 12, 24, 36 and 48 h (Day 14 only) post dose. Concurrently, blood samples were collected from 3 control rats/sex/timepoint at 0.5 and 2 h post dose. The study was performed at International Toxicology Research (ITR) and approved by their Animal Care Committee (ACC). Study no. 73762, August 2017.

#### 2.2.6. PK and Vaginal Biodistribution (BD) of Nano-Mupirocin

In a preliminary qualitative study, two female BALB/c mice were injected intra-peritoneally (IP) with LRPE-Nano-mupirocin (75 mg/kg). Vaginal swabs (COPAN, 160C, Murrieta, CA, USA) were taken 3.5 h later, with an additional swab from an untreated control mouse. Smears of the swabs were examined under a Nikon spinning disk confocal microscope for the presence of Nano-mupirocin liposomes fluorescently labelled with LRPE, using a 561 laser with CFI Plan-Apochromat Lambda x60 N.A. 0.95 objective.

Subsequently, Nano-mupirocin was administered at 50 mg/kg IP (dose calculated based on total mupirocin concentration in the formulation) to BALB/c female mice aged 6–7 weeks. At 1 h, 2 h, 4 h, 6 h, 8 h, 24 h post-dose, groups of 5 mice were subjected to vaginal swabbing, with the swabs immediately placed in tubes containing 2 mL acetonitrile. At each timepoint, mice were euthanized by CO_2_, with terminal blood collected from the retro-orbital sinus into K_3_EDTA tubes (Mini Collect, Greiner-bio-one, Kremsmünster, Austria) and centrifuged at 2000× *g* for 10 min to obtain plasma, which was stored at minus 80 °C pending analysis. This study was performed at the Hebrew University of Jerusalem and was approved by their ethics committee (approval MD-19-15898-3, 19 June 2019)

#### 2.2.7. Bioanalytical Methods

Bioanalytical testing was performed at two sites, with slightly different methods. Details are presented in the Supplementary Information.

In all pharmacokinetic studies, total mupirocin plasma concentrations were measured; the method did not distinguish between free, plasma bound and liposomal mupirocin. However, it is assumed that the great majority of the mupirocin recovered from the plasma is liposomal, because free drug is rapidly eliminated [9].

#### 2.2.8. Pharmacokinetic Analysis

Plasma concentrations at each time point were averaged, and PK parameters were calculated with Phoenix WinNonlin (CertaraTM, NJ, USA, Version 6.3), using a non-compartmental model and mean concentration data. C_max_ is as observed; C_0_ is the concentration estimated by the software at t = 0. The terminal slope (λ) was estimated by linear regression through the last >3 time points and was used to calculate the terminal t_1/2_. The area under the curve from dosing to the last time point (AUCz) was calculated by Linear Trapezoidal with Linear Interpolation; the AUC extrapolated to infinity (AUC∞) was calculated as AUCz + C_last_/λ, where C_last_ was the observed concentration at last time point. Plasma clearance (CL) was calculated as Dose /AUC∞ and the Volume of distribution (Vz) as Dose/λ × AUC∞.

#### 2.2.9. Necrotizing Fasciitis, Dose Response Study

The necrotizing fasciitis model was based on a published method [9,10,11,12,13,14]. Female Balb/c mice, 3–4 weeks old (Envigo, Ness Ziona, Israel) ~10 g, were injected subcutaneously with approximately 1 × 10^8^ CFU, M14 Group A Streptococcus (GAS). A single dose of Nano-mupirocin between1.1-57 mg/kg was administered IV 1 h after infection. Doses were calculated based on the total mupirocin concentration in the formulation. Mice were monitored for five days to evaluate disease severity and mortality. This study was performed at the Hebrew University of Jerusalem and approved by their ethics committee (approval MD-15-14369-5, 26 April 2015).

## 3. Results

### 3.1. Activity against Gram-Positive Bacteria

The activity of mupirocin and Nano-mupirocin were tested for 167 Gram-positive isolates at IHMA. A line listing of the isolates and MICs is presented in Supplementary Tables S1 and S2. Nano-mupirocin MICs were mostly 2- to 4-fold higher than those of free mupirocin for *S. aureus*, with modes at 0.5 and <0.25 µg/mL, respectively; MICs for *S. aureus* isolates with low-level mupirocin resistance were in the range of 16 μg/mL to above 64 μg/mL for both formulations (Table 1). MICs were unrelated to methicillin resistance status, and both mupirocin and Nano-mupirocin remained fully active against *S. aureus* resistant to vancomycin, daptomycin and linezolid (Supplementary Table S3). For *S. pyogenes* isolates, mupirocin and Nano-mupirocin generally both were active at ≤0.25 µg/mL and, for *S. pneumoniae*, at ≤0.5 and 2 µg/mL, respectively, with maximal MICs at 4 and 8 µg/mL (Table 2). Many pneumococci were resistant to penicillin, macrolides, and tetracycline and, for these, no cross-resistance to Nano-mupirocin was seen. 

Free mupirocin was tested against vancomycin-resistant enterococci at Public Health England (PHE) (Table 3). For *E. faecium*, most mupirocin MICs fell between 0.25–1 µg/mL, with 99.1% of isolates inhibited at 1 µg/mL and all at 2 µg/mL. Values for *E. faecalis* were much higher, clustering around 32–64 µg/mL. These patterns were confirmed by a smaller study at IHMA, which additionally found that Nano-mupirocin MICs were 2- to 4-fold above those of free mupirocin (Table 4). No cross-resistance to other agents was found in either study.

The bactericidal activity of mupirocin and Nano-mupirocin was tested against *S. aureus* and *Streptococcus* species as described in Table 5. Mupirocin and Nano-mupirocin displayed comparable MBCs against the isolates tested. For *S. pneumoniae* ATCC 49619 and the six *S. aureus* MRSA clinical isolates tested, the MBCs of mupirocin and Nano-mupirocin were identical or very similar to the MICs; however, MBCs for *S. aureus* ATCC 29213 and *S. pyogenes* were up to 64 times higher than the MIC. The reason for this difference is unknown.

### 3.2. Resistance Selection

#### 3.2.1. Gram-Positive Isolates

Resistance passage and single-step selection studies were undertaken for 3 *E. faecium* and 6 MRSA isolates, all at IHMA (Supplementary Tables S4–S6). Only one potential MRSA mutant was obtained with Nano-mupirocin in these passage studies and two with free mupirocin. Two of these three were confirmed to have reduced susceptibility, though MICs were still only 0.5–2 µg/mL. Three mutants were confirmed for mupirocin and four with Nano-mupirocin from the 3 *E. faecium* isolates during passage. MICs for these were in the range of 2–32 µg/mL, with cross resistance between the two formulations.

During single step studies with free mupirocin (Supplementary Tables S7–S9), no mutants were confirmed for MRSA and *E. faecium*, indicating mutation frequencies below the detection limits of <1.25 × 10^−9^ to <6.76 × 10^−10^. For Nano-mupirocin, 19 mutants were confirmed: MICs for both mupirocin and Nano-mupirocin for these were increased by 8-128-fold, and frequencies were in the range of 3.63 × 10^−8^ to 7.28 × 10^−10^. The higher rates with Nano-mupirocin may be an artifact of the method used to disperse the drug in the MHA (see Section 2.2.4). Free mupirocin was added to molten MHA then poured into plates, allowing homogenous distribution whereas, owing to the heat-lability of liposomes, Nano-mupirocin was spread on solidified MHA and allowed to diffuse into this medium, likely resulting in a less even distribution.

#### 3.2.2. Resistance Selection with *N. gonorrhoeae*

Three *N. gonorrhoeae* isolates were tested. Their tolerance to both mupirocin forms (free and liposomal) increased by 4- to 8-fold over 15 days of passaging. However, only one mutant was confirmed for Nano-mupirocin, and the MIC for this organism, after 5 non-selective subcultures, remained only 0.12 µg/mL, compared with a starting MIC of 0.03 µg/mL, which corresponds to the mode for the species [15]. No mutants were confirmed for free mupirocin in the passage study, and none were obtained with either formulation in the single-step study (Supplementary Tables S10–S12).

### 3.3. Pharmacokinetic (PK) Study

**Figure 1:**
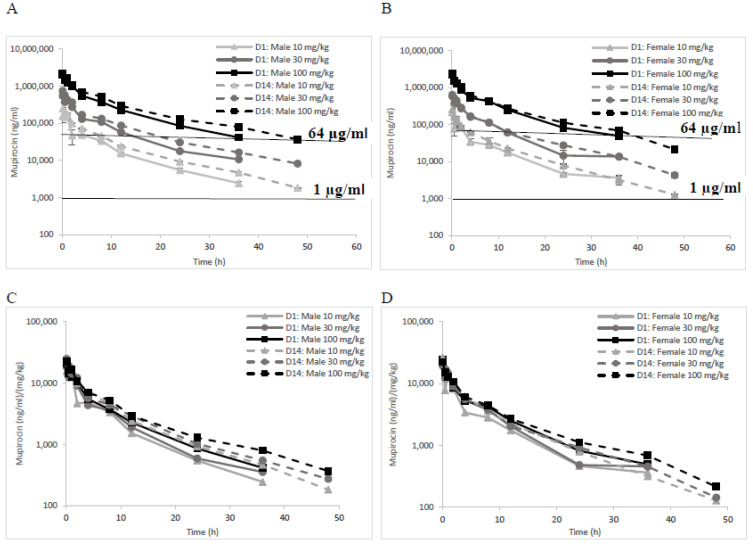
Total mupirocin; (liposome-encapsulated plus non-liposomal drug plus plasma-protein-bound drug) concentration (ng/mL) following IV administration of 10, 30 and 100 mg/kg Nano-mupirocin to male (**A**) and female (**B**) rats on days 1 and 14. (**C**,**D**) represent normalization of the profiles to the doses (*n* = 3, mean ± SE). The horizontal line of 1 µg/mL in (**A**,**B**) corresponds to the MIC for most Gram-positive isolates (except *E. faecalis*), and the 64 µg/mL line to the maximal MIC for MRSA with low-level mutational-type mupirocin resistance.

This study tested the toxicology and PK of increasing (10, 30 and 100 mg/kg) IV Nano-mupirocin doses administered three times a week for two weeks to male and female rats (as detailed in Section 2.2.5). The pharmacokinetic profiles obtained are depicted in Figure 1, where the plasma concentrations represent total mupirocin (i.e., liposome-encapsulated plus non-liposomal drug plus plasma-protein-bound drug). It is assumed that most detected drug is liposomal (Nano-mupirocin), as free mupirocin is rapidly metabolized and cleared [9].

PK profiles were similar for male and female rats on days 1 and 14. Quantifiable mupirocin was still detectable at the last bleeding (36 and 48 h post dose, on days 1 and 14, respectively). 

Following the first administration, and contingent on the dosage, the mean C_max_ on day 1 ranged from 161 to 2087 µg/mL for male rats and from 216 to 2400 µg/mL for females. Mupirocin plasma concentrations increased proportionally with dose (Figure 1C,D), as did AUC_INF_ (Supplementary Table S13); the deviation of each parameter, normalized to dose, from the average was <15%. C_max_ and AUC at later intervals in the 2-week treatment period remained comparable to the values obtained on Day 1, suggesting that there was no appreciable accumulation.

The estimated mean t_1/2_ ranged from 8.33 to 9.78 h for males and between 6.76 and 9.04 h for females, remaining similar between Days 1 and 14. Mean total body clearance rates (Cl) ranged from 9.44 to 12.20 mL/h/kg for males and from 9.22 to 12.38 mL/h/kg for females, again remaining similar on Days 1 and 14. The volume of distribution (Vz) ranged from 131 to 160 mL/kg and from 100 to 161 mL/kg for males and females, respectively, likewise remaining similar on Days 1 and 14. The pharmacokinetic parameters are summarized in Table 6.

**Figure 2:**
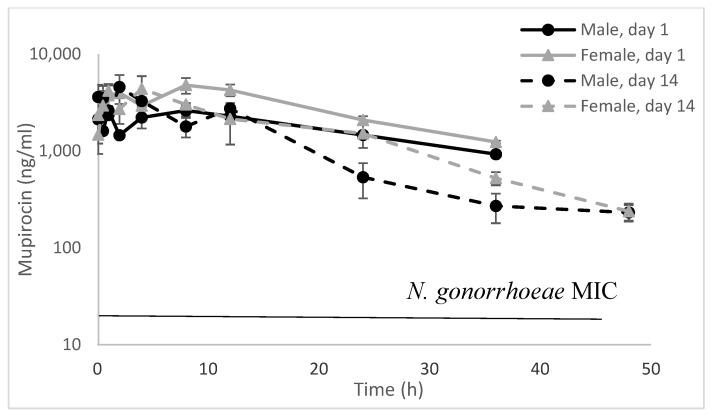
Total mupirocin (liposome-encapsulated plus non-liposomal drug plus plasma-protein-bound drug) concentration (ng/mL) following IM administration of 2.6 mg Nano-mupirocin to male and female rats on days 1 and 14. (*n* = 3, mean ± SE).

Following Day 1 IM administration of 2.6 mg (~10.5 mg/kg) Nano-mupirocin, plasma levels over 12–24 h after injection ranged from 0.929 to 2.620 µg/mL for male rats, and from 1.240 to 4.787 µg/mL for females (Figure 2 and Table 7). Similar values were seen on Day 14. Total exposures on Days 1 and 14 was also similar, suggesting no appreciable accumulation (Table 7). Mean bioavailability following IM injection (% F) was 8 and 14% in males and females, respectively, after the first injection, and 5 and 7%, respectively, after the final injection. 

.

Toxicology details are beyond the scope of the present paper. However, there were no adverse findings, and the no-observable-adverse-effect level (NOAEL) was taken as the highest dose level assessed—100 mg/kg/dose.

### 3.4. Nano-mupirocin Biodistribution into Murine Vaginal Secretions

**Figure 3:**
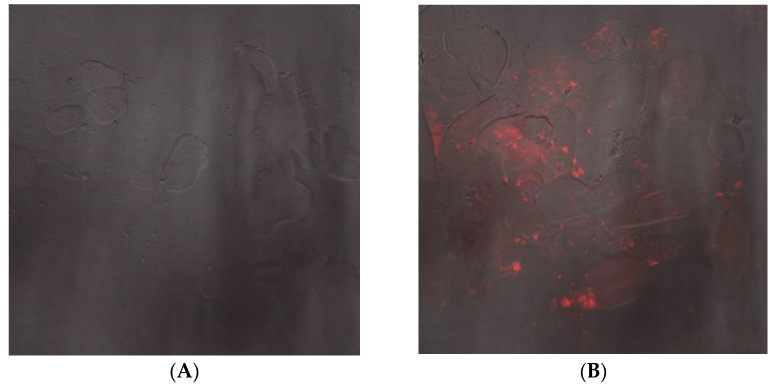

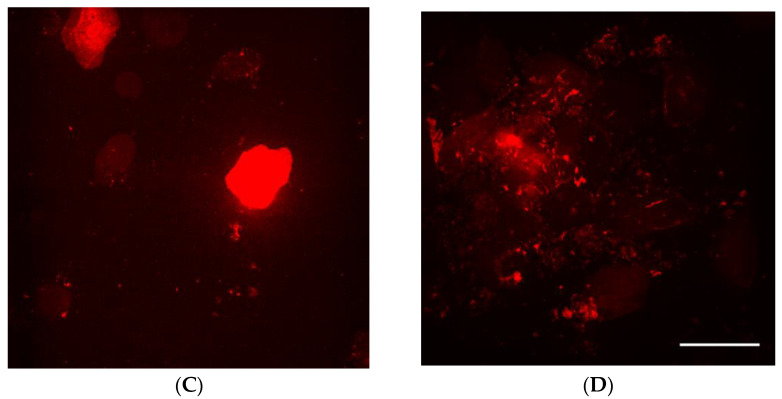
Fluorescence microscopy of vaginal smears. (**A**,**B**) are overlays of Differential interference contrast (DIC) and Fluorescent Light. (**A**), un-treated mice; (**B**), mice treated with LRPE-Nano-mupirocin; (**C**,**D**), smears of LRPE-Nano-mupirocin observed under fluorescent light. Scale bar = 50 μm.

To assess the potential of Nano-mupirocin for the treatment of gonorrhea, its biodistribution (BD) into murine vaginal secretions was determined in healthy mice. Two methods were used. The first was qualitative: female mice were injected with LRPE-Nano-mupirocin IP, and 3.5 h after injection, vaginal swabs were taken, along with an additional swab from an untreated mouse. Smears of these swabs were observed under a spinning disk confocal microscope (Figure 3), qualitatively revealing substantial fluorescence in the treated mice. A Supplementary short movie illustrates moving fluorescent commensal bacteria that have taken up the fluorescent liposomes, confirming that these reach the vaginal secretions and can interact with bacteria.

**Figure 4:**
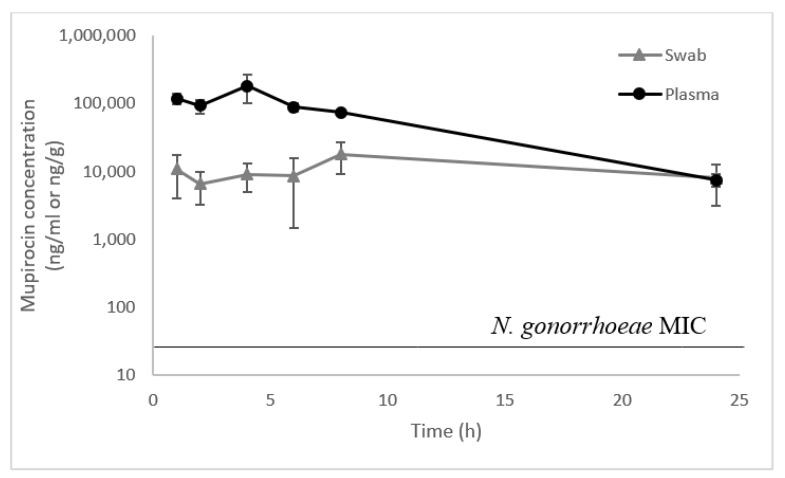
Mupirocin concentration (free (non-liposomal) plus liposomal) in vaginal secretions (ng/g) and plasma (ng/mL) following IP administration of Nano-mupirocin 50 mg/kg (mean ± SE). (*n* = 5 for swab samples and *n* = 4 for plasma samples).

The second study measured the amount of mupirocin that reached the vaginal secretion (Figure 4). The maximum plasma concentration after a 50 mg/kg dosage was 182 µg/mL, 4 h after administration. Concentrations in vaginal secretions averaged 11 µg/g, 1 h after administration and 8 µg/g, 24 h after administration; they varied greatly among animals but even the lowest concentrations (with the exception of one sample below limit of quantitation) were above the MIC_90_ for *N. gonorrhoeae* (0.03 µg/mL) (15). The vaginal secretion AUCz was 284 µg × h/g, amounting to 19% of plasma AUCz of 1532 µg × h/mL.

### 3.5. Dose Response Study in Necrotizing Fasciitis

**Figure 5:**
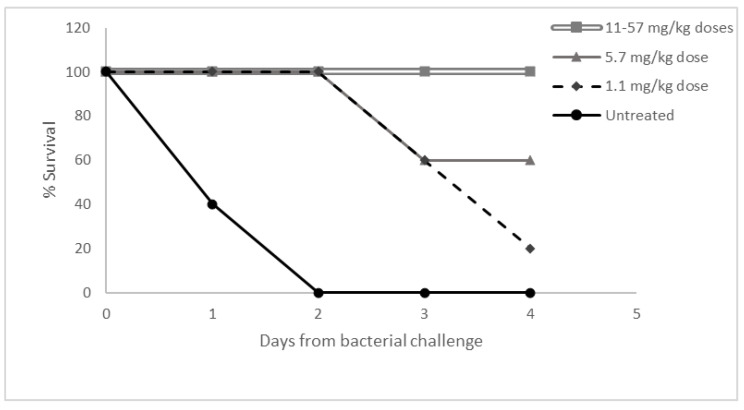
Dose response to Nano-mupirocin in a murine necrotizing fasciitis model.

Nano-mupirocin previously showed efficacy in a mouse necrotizing fasciitis model with a group A Streptococcus (GAS), [9] and a further dose–response study was performed with the same model. A single 1.1–57 mg/kg dose of Nano-mupirocin was administered IV 1 h after infection, with survival followed for 5 days (Figure 5). In the control group, 60% mortality occurred on the first day of infection, with all animals dying within 48 h. At the lowest Nano-mupirocin dose (1.1 mg/kg), mortality only started 3 days after infection, two days later than the control. At higher doses (11–57 mg/kg), no mortality occurred.

These results are in keeping with previous studies showing that mupirocin has time-dependent bactericidal activity [16], and the view that a cidal concentration must be maintained for over 4 h. Thus, complete survival was achieved with a single 11 mg/kg dose which, based on our previous PK data [9], is predicted to result in a ~20 µg/mL plasma concentration even 4 h after administration. At a 10-fold lower dose (1.1 mg/kg), the predicted concentration at 4 h (2 µg/mL) bordered the MBCs, which were raised compared with MICs for *S. pyogenes* (see Table 5), perhaps explaining why some delayed mortality was seen.

## 4. Discussion

Mupirocin has a long history of use for superficial staphylococcal skin infections and for elimination of nasal MRSA. Parenteral Nano-mupirocin, being protected from rapid metabolism, opens the novel possibility of use against deep infections and against other pathogens. Nano-mupirocin is a stable product with a loading stability of at least 2 years at 4 °C (not shown).

MICs of Nano-mupirocin were mostly 2- to 4-fold above those of the free drug. This differential is surprisingly small, given that the in vitro release of free drugs from the liposomes is slow [17]. The explanation, based on previous results, is that the intact Nano-mupirocin interacts directly with *S. aureus* and does not require drug release to achieve an antibacterial effect [10]. 

Mupirocin’s unique mode of action suggests that it should retain activity against otherwise resistant strains, and this indeed was seen, with activity confirmed against MRSA resistant to vancomycin, daptomycin and linezolid, vancomycin-resistant *E*. *faecium*, and, previously, against *N. gonorrhoeae* isolates resistant to extended-spectrum cephalosporins [15].

The rat pharmacokinetic study performed here demonstrated a linear increase in exposure with dose, with neither accumulation nor faster clearance upon repeated administration. Exposures in this study can usefully be compared with MICs of key pathogens, as represented by the two horizontal lines in Figure 1A,B. The 1 µg/mL line corresponds to the MIC for most Gram-positive isolates (except *E*. *faecalis*), and the 64 µg/mL line to the maximal MIC for MRSA with low-level mutational-type mupirocin resistance [18]. Concentrations > 1 µg/mL were achieved for >48 h even after the lowest dose administered (10 mg/kg, equivalent to 97 mg for a 60 kg human [19]). Mupirocin has time-dependent bactericidal activity [16] and, in our previous in vitro study, achieved complete clearance of bacteria in three independent experiments, during 4 h of incubation in the presence of plasma [10]. Here, 10, 30, and 100 mg/kg doses of Nano-mupirocin to male rats gave plasma drug concentrations of 49, 131, and 552 µg/mL, respectively, at 4 h post-dose, with all these levels remaining far above MBC values (Table 4).

IM antibiotic administration is preferred for some infections, notably gonorrhea. Rat plasma concentrations after IM administration of Nano-mupirocin greatly exceeded the MIC_90_ of 0.03 µg/mL for *N. gonorrhoeae* [15] at all time points tested. However, the pharmacodynamic drivers in gonorrhea are poorly defined, and plasma concentrations may not reflect concentrations at disease sites [20]. Moreover, the female mouse model of gonococcal infection developed by Prof. Jerse’s laboratory [21] proved to be unsuitable for testing Nano-mupirocin due to requiring pre-treatment of the animals with estrogen. This causes thickening of the vaginal mucosa by stimulating the proliferation of epithelial cells [21,22] which then appears to serve as a barrier for the nano-liposomes. An in-house study showed that the levels of mupirocin achieved in the vaginal secretions of estrogen-treated mice were much lower than in un-treated animals, and that the secretions were denser and thicker (not shown). Therefore, we measured drug concentrations in the vaginal secretions, showing that, over the 24 h after injection, mupirocin was present at concentrations much above the MIC_90_, ranging from 11 µg/g 1 h after injection to 8 µg/g, 24 h after injection and corresponding to 267–367 times the MIC_90_. Nano-mupirocin addresses many of the criteria of a Consensus Target Product Profile recently published by a gonorrhea expert group [23] including: (i) activity against *Mycoplasma genitalium* [24]; (ii) activity against cephalosporin- and macrolide- resistant gonococci [15]; (iii) intracellular activity [10]; (iv) lack of cross-resistance; and (v) suitability for IM injection.

Low-level mupirocin resistance in staphylococci is well-recognized. It arises by mutation and may reflect high local usage of topical mupirocin [18]. In the light of this concern, we undertook mutation frequency and 15-day passage studies. Very little resistance emerged, particularly for MRSA and *N. gonorrhoeae*. More mutants were selected with Nano-mupirocin than with free mupirocin, which may be a result of technical differences related to drug dispersion in MHA (see Section 3.1). High drug concentrations at infections sites should militate against selection in vivo [9,10], with activity predicted (above) even against *S. aureus* with low-level mutational resistance. Moreover, the reported association [18] between mupirocin use and resistance prevalence may reflect the spread of resistant strains, rather than repeated de novo selection.

Nano-mupirocin has potential for multiple indications where the causative bacteria are typically susceptible. These include gonorrhoea as well as deep infections such as pneumonia and osteomyelitis. Patients with these infections would benefit from a safe drug that is distributed at the infection site [10]. Nano-mupirocin uses a known antibiotic and PEGylated nano-liposomes identical in lipid composition and size to those of Doxil^®^ [25], which has been used in >700,000 cancer patients. The PK and toxicity of free mupirocin were evaluated after IV injection in healthy volunteers up to a dose of 252 mg/person, with good tolerability [26]. Accordingly, Nano-mupirocin should be seen as a formulation with considerable potential and low development risks.

## Figures and Tables

**Table 1:** Mupirocin and Nano-mupirocin MICs for methicillin-susceptible *Staphylococcus aureus* (MSSA) and methicillin-resistant *Staphylococcus aureus* (MRSA) isolates.

	No. Isolates with Indicated Mupirocin MIC (MSSA, MRSA)											
No. isolates with indicated Nano-mupirocin MIC (MSSA, MRSA)	µg/mL	0.12	0.25	0.5	1	2	4	8	16	32	64	>64
	0.12	1, 3										
	0.25	6, 5	7, 1									
	0.5	7, 6	28, 18	0, 1								
	1		1, 7	0, 1								
	2											
	4											
	8											
	16							0, 1				
	32								0, 2			
	64									0, 1		
	>64								0, 1	0, 1		1, 3

Of the 51 MRSA isolates, 16% were vancomycin resistant, 24% daptomycin resistant, and 18% linezolid resistant.

**Table 2:** Mupirocin and Nano-mupirocin MICs for *S. pneumoniae* (*n* = 25) and *S. pyogenes* isolates (*n* = 26).

	No. Isolates with Indicated Mupirocin MIC (*S. pneumoniae, S. pyogenes*)								
		≤ 0.06	0.06	0.12	0.25	0.5	1	2	4
No. isolates with indicated Nano-mupirocin MIC (*S. pneumoniae, S. pyogenes*)	≤0.06								
	0.06	0, 1			0, 1				
	0.12			0, 13	0, 3				
	0.25			0, 3	3, 3				
	0.5				3, 1	1, 0	1, 0		
	1				1, 0	6, 0			
	2					1, 0			
	4						1, 1	2, 0	
	8							2, 0	4, 0

Among the 25 *S. pneumoniae* isolates, 44% were penicillin resistant, 52% erythromycin resistant, and 56% tetracycline resistant.

**Table 3:** Mupirocin activity against vancomycin-resistant *E. faecium* and *E. faecalis* isolates: PHE data.

	No. Isolates with MIC (µg/mL)												
	0.125	0.25	0.5	1	2	4	8	16	32	64	128	256	≥512
*E. faecium* VanR group (*n* = 115)													
Mupirocin	7	73	9	25	1								
Vancomycin	1		2							112			
Linezolid				67	32		6	8	2				
*E. faecalis* VanR group (*n* = 101)													
Mupirocin							1	12	42	42	3		1
Vancomycin				5	1	5	2	3	1	84			
Linezolid				83	17			1					

**Table 4:** Mupirocin and Nano-mupirocin MICS for *E. faecium* and *E. faecalis*.

	MIC (µg/mL)									
Organism No.	Mupirocin	Nano-Mupirocin	Vancomycin	Linezolid	Daptomycin	Penicillin	Erythromycin	Tetracycline	Levofloxacin	Trimethoprim/
										Sulfamethoxazole
*E. faecium*										
1146992	1	4	>16	>8	2	>16	>4	4	>4	>2
1533772	0.5	2	>16	>8	4	>16	2	32	>4	>2
1765156	1	2	>16	8	4	>16	>4	0.5	>4	>2
1766256	1	2	>16	4	4	>16	>4	16	>4	>2
1602010	1	2	1	2	4	>16	2	>32	>4	>2
1602013	1	2	1	2	4	>16	2	>32	>4	>2
1765227	0.5	1	2	2	0.5	0.12	>4	>32	2	≤0.06
*E. faecalis*										
862935	64	>64	1	2	1	2	0.25	>32	1	≤0.06
1569172	>64	>64	1	2	1	2	2	>32	1	≤0.06
1606748	32	>64	1	2	2	2	0.25	16	2	≤0.06
1765036	64	>64	0.5	1	1	2	0.5	0.5	1	≤0.06
860769	32	>64	1	2	2	2	>4	32	1	>2
1766601	64	>64	>16	2	0.5	2	>4	>32	>4	>2
1766602	64	>64	>16	2	2	8	>4	>32	>4	>2

**Table 5:** MBC vs. MIC of mupirocin and Nano-mupirocin for reference strains of *S. aureus* and *Streptococcus* spp.

			MIC (µg/mL)		MBC (µg/mL)	
Organism	Organism No.	Resistance	Mupirocin	Nano-Mupirocin	Mupirocin	Nano-Mupirocin
*S. aureus*	ATCC 29213	*NA*	0.12	0.5	32	32
*S. aureus*	ATCC 29213	*NA*	0.25	0.5	16	16
MRSA	649380	*NA*	0.12	0.5	0.12	1
MRSA	649390	*NA*	0.25	1	0.25	2
MRSA	1308254	Daptomycin non-susceptible	0.12	0.5	0.25	2
MRSA	672231	Vancomycin resistant	0.06	0.25	0.12	1
MRSA	672233	Vancomycin resistant	0.06	0.5	0.12	1
MRSA	672232	Vancomycin resistant	0.12	0.5	0.5	4
*S. pneumoniae*	ATCC 49619	*NA*	0.12	0.25	0.25	1
*S. pneumoniae*	ATCC 49619	*NA*	0.25	0.5	0.5	1
*S. pyogenes*	1262561	Macrolide resistant	0.25	0.5	16	32
*S. pyogenes*	1426536	Macrolide resistant	0.03	0.12	8	8
*S. pyogenes*	1440834	Macrolide resistant	0.12	0.12	4	4

NA—Not applicable. Abbreviations: NA.

**Table 6:** Pharmacokinetic parameters following IV administration of 10, 30, and 100 mg/kg Nano-mupirocin to rats.

	T_1/2_	T_max_	C_max_	C_0_	AUC_0_Tlast_	AUC_INF_	V_z_	Cl
	(h)	(h)	(µg/mL)	(µg/mL)	(h × µg/mL)	(h × µg/mL)	(mL/kg)	(mL/h/kg)
**Day 1**								
**Male**								
10 mg/kg	9.06	1.00	161	160	788	820	160	12.20
30 mg/kg	8.33	0.08	551	596	2617	2745	131	10.93
100 mg/kg	9.78	0.08	2087	2246	10,010	10,596	133	9.44
**Female**								
10 mg/kg	9.04	0.08	216	265	761	808	161	12.38
30 mg/kg	6.76	0.08	582	639	2784	2917	100	10.29
100 mg/kg	8.89	0.08	2400	2610	10,218	10,848	118	9.22
**Day 14**								
**Male**								
10 mg/kg	9.87	0.08	248	266	1208	1234	115	8.11
30 mg/kg	12.59	0.08	746	787	3714	3863	141	7.77
100 mg/kg	12.41	0.08	2233	2381	13,554	14,213	126	7.04
**Female**								
10 mg/kg	9.13	0.08	263	288	1036	1053	125	9.50
30 mg/kg	8.72	0.08	636	664	3089	3143	120	9.55
100 mg/kg	9.54	0.08	2227	2401	11,980	12,273	112	8.15

**Table 7:** Pharmacokinetic parameters following IM administration of 2.6 mg Nano-mupirocin per rat.

	T_1/2_	T_max_	C_max_	AUC_0_Tlast_	AUC_INF_	%F ^a^
	(h)	(h)	(µg/mL)	(h × µg/mL)	(h × µg/mL)	
**Day 1**						
Male	18.52	1.00	2.62	63.96	88.78	8.12
Female	13.45	8.00	4.79	105.68	129.75	13.89
**Day 14**						
Male	10.68	2.00	4.58	61.22	64.79	5.07
Female	9.01	4.00	4.39	77.19	80.30	7.45

a-%F was calculated by the following equation: $\%\;F=\frac{\mathrm{AUC}\;0\_\mathrm{Tlast}\;\mathrm{after}\;\mathrm{IM}\;\mathrm{administration}}{\mathrm{AUC}\;0\_\mathrm{Tlast}\;\mathrm{after}\;\mathrm{IV}\;10\;\mathrm{mg}/\mathrm{kg}\;\mathrm{administration}}\times 100$.

## Data Availability

Not applicable.

## Supplementary Materials

The following are available online at https://www.mdpi.com/article/10.3390/pharmaceutics13122186/s1. Table S1. Line listing of the 167 Gram-positive isolates tested. Table S2. Line listing of MIC data for 167 Gram-positive isolates. Table S3. Mupirocin and Nano-mupirocin MICs for MRSA isolates resistant to one or more of vancomycin, daptomycin or linezolid. Table S4. MICs of Nano-mupirocin, mupirocin and rifampicin during passage/multi-step selection for nine Gram-positive clinical isolates. Table S5. MICs for potential mutants obtained in multi-step selection, as re-tested after five days of subculture on drug- free agar. Table S6. Line listing of agar MIC data for Nano-mupirocin, mupirocin, and rifampicin against nine Gram-positive clinical isolates, as tested at different inocula, and for two QC strains. Table S7. Line listing of single-step spontaneous mutation frequency data for nine Gram-positive isolates tested with Nano-mupirocin, mupirocin, and rifampicin. Table S8. Line listing of MICs for variants selected in spontaneous mutation frequency experiments. Table S9. MICs of Nano-mupirocin, mupirocin and tetracycline for three *N. gonorrhoea* clinical isolates during multi-step passaging study. Table S10. MIC data for potential mutants *of N. gonorrhoea* as re-tested after five days subculture on drug-free agar. Table S11. Line listing of MIC data for three *N. gonorrhoea* clinical isolates tested at different inocula and for one QC strain. Table S12. Line listing of spontaneous mutation frequency (SMF) data for *N. gonorrhoea.* Table S13. Exposure parameters (Cmax and AUCINF) for Nano-mupirocin in rats, according to dose and as compared with mean values. Video S1. One minute time laps movie was taken for smear of a vaginal swab from a mouse injected with LRPE-Nano-mupirocin, examined under Nikon spinning disk confocal microscope. The arrows in the movie show moving fluorescent commensal bacteria that have taken up the fluorescent liposomes. Scale bar = 10 µm.
