# Supplementary material for: Therapeutic Potential of Injectable Nano-Mupirocin Liposomes for Infections Involving Multidrug-Resistant Bacteria
Source: Pharmaceutics. 2021 Dec 17;13(12):2186. doi: 10.3390/pharmaceutics13122186 (PMC8706398; doi:10.3390/pharmaceutics13122186)
Supplement: Supplementary file 1 [file pharmaceutics-13-02186-s001.zip › pharmaceutics-1503216-Supplementary materials/pharmaceutics-1503216-Supplementary materials.pdf]

# Supplementary Materials: Therapeutic Potential of Injectable Nano-Mupirocin Liposomes for Infections Involving Multidrug-Resistant Bacteria

**Ahuva Cern, Yaelle Bavli, Atara Hod, Daniel Zilbersheid, Shazad Mushtaq, Ayelet Michael-Gayego, Dinorah Barasch, Yael Feinstein Rotkopf, Allon E. Moses, David M Livermore and Yechezkel Barenholz**

Table S1. Line listing of the 167 Gram-positive isolates tested

| # | Organism No | Organism | Country | Year Collected | Body Location | Expected Resistance | Final Resistance |
|---|---|---|---|---|---|---|---|
| 1 | 626678 | *S. aureus*, MRSA | United States | 2010 | Bodily Fluids: Synovial | DAP R | VAN S / DAP NS / LNZ S |
| 2 | 410696 | *S. aureus*, MRSA | United States | 2008 | CVS: Blood | DAP R | VAN S / DAP NS / LNZ S |
| 3 | 651970 | *S. aureus*, MRSA | Canada | 2010 | INT: Wound | LNZ R | VAN S / DAP S / LNZ R |
| 4 | 672232 | *S. aureus*, MRSA | United States | 2010 | Unknown | VAN R | VAN R / DAP S / LNZ S |
| 5 | 1146992 | *E. faecium* | Austria | 2014 | INT: Wound | LNZ R / VAN R | LNZ R / VAN R |
| 6 | 1180874 | *S. aureus*, MRSA | Spain | 2014 | GI: Liver | LNZ R | VAN S / DAP S / LNZ R |
| 7 | 746091 | *S. aureus*, MRSA | Spain | 2011 | Respiratory: Bronchials | LNZ R | VAN S / DAP S / LNZ S |
| 8 | 817763 | *S. aureus*, MRSA | United States | 2012 | Respiratory: Bronchials | LNZ R | VAN S / DAP S / LNZ R |
| 9 | 651971 | *S. aureus*, MRSA | Canada | 2010 | INT: Wound | LNZ R | VAN S / DAP S / LNZ R |
| 10 | 878234 | *S. aureus*, MRSA | Spain | 2012 | INT: Wound | DAP R | VAN S / DAP NS / LNZ S |
| 11 | 649380 | *S. aureus*, MRSA | United States | 1997 | Blood stream | VAN I | VAN I / DAP S / LNZ S |
| 12 | 649390 | *S. aureus*, MRSA | United States | 2000 | Bone/joint | VAN I | VAN I / DAP S / LNZ S |
| 13 | 649391 | *S. aureus*, MRSA | United States | 2000 | Wound | VAN I | VAN I / DAP S / LNZ S |
| 14 | 1308248 | *S. aureus*, MRSA | United States | 2000 | Cerebrospinal fluid | VAN I | VAN I / DAP NS / LNZ S |
| 15 | 1308252 | *S. aureus*, MRSA | United States | 2000 | Bile | VAN I | VAN S / DAP S / LNZ S |
| 16 | 1308253 | *S. aureus*, MRSA | Brazil | 1998 | Wound | VAN I | VAN S / DAP S / LNZ S |
| 17 | 1308254 | *S. aureus*, MRSA | Brazil | 1999 | Wound | VAN I | VAN I / DAP NS / LNZ S |
| 18 | 672230 | *S. aureus*, MRSA | United States | 2010 | Unknown | VAN R | VAN R / DAP S / LNZ S |
| 19 | 672231 | *S. aureus*, MRSA | United States | 2010 | Unknown | VAN R | VAN R / DAP S / LNZ S |
| 20 | 672233 | *S. aureus*, MRSA | United States | 2010 | Unknown | VAN R | VAN R / DAP S / LNZ S |
| 21 | 672234 | *S. aureus*, MRSA | United States | 2010 | Unknown | VAN R | VAN R / DAP S / LNZ S |
| 22 | 672235 | *S. aureus*, MRSA | United States | 2010 | Unknown | VAN R | VAN R / DAP S / LNZ S |
| 23 | 1308256 | *S. aureus*, MRSA | United States | 2009 | Wound | VAN R | VAN R / DAP S / LNZ S |
| 24 | 1308258 | *S. aureus*, MRSA | United States | 2010 | Wound | VAN R | VAN R / DAP S / LNZ S |
| 25 | 649401 | *S. aureus*, MRSA | United States | 2001 | Peritonitis | LNZ R | VAN S / DAP S / LNZ R |
| 26 | 649402 | *S. aureus*, MRSA | United States | 2001 | Peritonitis | LNZ R | VAN S / DAP S / LNZ R |
| 27 | 649404 | *S. aureus*, MRSA | United States | 2002 | Sputum | LNZ R | VAN S / DAP S / LNZ R |
| 28 | 309191 | *S. aureus*, MRSA | Belgium | 2007 | HEENT: Nose | LNZ R / VAN R | VAN S / DAP S / LNZ S |
| 29 | 114489 | *S. aureus*, MRSA | Italy | 2001 | CVS: Blood | LNZ R / VAN R | VAN S / DAP S / LNZ S |
| 30 | 860769 | *E. faecalis* | Colombia | 2012 | Bodily Fluids: Peritoneal | LNZ R / VAN R | LNZ S / VAN S / DAP NS |
| 31 | 862935 | *E. faecalis* | Turkey | 2012 | GU: Urine | LNZ R / VAN R | LNZ S / VAN S |
| 32 | 925016 | *S. aureus*, MRSA | Germany | 2013 | INT: Wound | DAP R | VAN S / DAP S / LNZ S |
| 33 | 943616 | *S. aureus*, MRSA | Austria | 2013 | INT: Skin Ulcer | DAP R | VAN S / DAP NS / LNZ S |
| 34 | 1018136 | *S. aureus*, MRSA | Spain | 2013 | INT: Wound | DAP R | VAN S / DAP NS / LNZ S |

| # | Organism No | Organism | Country | Year Collected | Body Location | Expected Resistance | Final Resistance |
|---|---|---|---|---|---|---|---|
| 35 | 1018140 | *S. aureus,* MRSA | Spain | 2013 | INT: Wound | DAP R | VAN S / DAP NS / LNZ S |
| 36 | 927059 | *S. aureus,* MRSA | Turkey | 2013 | INT: Abscess | DAP R | VAN S / DAP NS / LNZ S |
| 37 | 927060 | *S. aureus,* MRSA | Turkey | 2013 | INT: Wound | DAP R | VAN S / DAP NS / LNZ S |
| 38 | 927061 | *S. aureus,* MRSA | Turkey | 2013 | INT: Wound | DAP R | VAN S / DAP NS / LNZ S |
| 39 | 928778 | *S. aureus,* MRSA | Spain | 2013 | INT: Wound | LNZ R | VAN S / DAP S / LNZ R |
| 40 | 1246376 | *S. aureus,* MSSA | United States | 2015 | INT: Wound | - | VAN S / DAP S / LNZ S |
| 41 | 1268522 | *S. aureus,* MSSA | Austria | 2015 | INT: Wound | - | VAN S / DAP S / LNZ S |
| 42 | 1268530 | *S. aureus,* MRSA | Austria | 2015 | INT: Decubitus | - | VAN S / DAP S / LNZ S |
| 43 | 1268531 | *S. aureus,* MRSA | Austria | 2015 | INT: Skin Ulcer | - | VAN S / DAP S / LNZ S |
| 44 | 1268648 | *S. aureus,* MRSA | Austria | 2015 | INT: Wound | - | VAN S / DAP S / LNZ S |
| 45 | 1272493 | *S. aureus,* MSSA | United States | 2015 | INT: Abscess | - | VAN S / DAP S / LNZ S |
| 46 | 1282516 | *S. aureus,* MSSA | Canada | 2015 | INT: Wound | - | VAN S / DAP S / LNZ S |
| 47 | 1276929 | *S. aureus,* MSSA | France | 2015 | INT: Abscess | - | VAN S / DAP S / LNZ S |
| 48 | 1335968 | *S. aureus,* MSSA | United Kingdom | 2015 | INT: Wound | - | VAN S / DAP S / LNZ S |
| 49 | 1285320 | *S. aureus,* MRSA | United States | 2015 | INT: Wound | - | VAN S / DAP NS / LNZ S |
| 50 | 1259571 | *S. aureus,* MRSA | Denmark | 2015 | INT: Abscess | - | VAN S / DAP S / LNZ S |
| 51 | 1336167 | *S. aureus,* MSSA | United States | 2015 | INT: Burn | - | VAN S / DAP S / LNZ S |
| 52 | 1267763 | *S. aureus,* MSSA | United States | 2015 | INT: Abscess | - | VAN S / DAP S / LNZ S |
| 53 | 1435278 | *S. aureus,* MSSA | United States | 2016 | INT: Skin Ulcer | - | VAN S / DAP S / LNZ S |
| 54 | 1428168 | *S. aureus,* MSSA | United States | 2016 | INT: Wound | - | VAN S / DAP S / LNZ S |
| 55 | 1475091 | *S. aureus,* MSSA | France | 2016 | INT: Wound | - | VAN S / DAP S / LNZ S |
| 56 | 1475093 | *S. aureus,* MSSA | France | 2016 | INT: Wound | - | VAN S / DAP S / LNZ S |
| 57 | 1518939 | *S. aureus,* MSSA | Czech Republic | 2016 | INT: Wound | - | VAN S / DAP S / LNZ S |
| 58 | 1518943 | *S. aureus,* MSSA | Czech Republic | 2016 | INT: Wound | - | VAN S / DAP S / LNZ S |
| 59 | 1528968 | *S. aureus,* MRSA | Belgium | 2016 | INT: Other | - | VAN S / DAP S / LNZ S |
| 60 | 1481133 | *S. aureus,* MRSA | Czech Republic | 2016 | INT: Wound | - | VAN S / DAP S / LNZ S |
| 61 | 1502818 | *S. aureus,* MSSA | Italy | 2016 | INT: Wound | - | VAN S / DAP S / LNZ S |
| 62 | 1502830 | *S. aureus,* MSSA | Italy | 2016 | INT: Wound | - | VAN S / DAP S / LNZ S |
| 63 | 1502834 | *S. aureus,* MSSA | Italy | 2016 | INT: Wound | - | VAN S / DAP S / LNZ S |
| 64 | 1540234 | *S. aureus,* MSSA | Germany | 2016 | INT: Wound | - | VAN S / DAP S / LNZ S |
| 65 | 1540236 | *S. aureus,* MSSA | Germany | 2016 | INT: Wound | - | VAN S / DAP S / LNZ S |
| 66 | 1540244 | *S. aureus,* MSSA | Germany | 2016 | INT: Wound | - | VAN S / DAP S / LNZ S |
| 67 | 1503418 | *S. aureus,* MSSA | Germany | 2016 | INT: Wound | - | VAN S / DAP S / LNZ S |
| 68 | 1503430 | *S. aureus,* MSSA | Germany | 2016 | INT: Wound | - | VAN S / DAP S / LNZ S |
| 69 | 1503437 | *S. aureus,* MSSA | Germany | 2016 | INT: Abscess | - | VAN S / DAP S / LNZ S |
| 70 | 1503428 | *S. aureus,* MSSA | Germany | 2016 | INT: Abscess | - | VAN S / DAP S / LNZ S |
| 71 | 1479809 | *S. aureus,* MRSA | France | 2016 | INT: Abscess | - | VAN S / DAP S / LNZ S |
| 72 | 1551440 | *S. aureus,* MSSA | Greece | 2016 | Bodily Fluids: Abscess/Pus | - | VAN S / DAP S / LNZ S |
| 73 | 1284277 | *S. aureus,* MSSA | France | 2015 | INT: Abscess | - | VAN S / DAP S / LNZ S |
| 74 | 1247465 | *S. aureus,* MSSA | United States | 2015 | INT: Wound | - | VAN S / DAP S / LNZ S |
| 75 | 1303514 | *S. aureus,* MRSA | Belgium | 2015 | INT: Wound | - | VAN S / DAP S / LNZ S |
| 76 | 1260284 | *S. aureus,* MSSA | Czech Republic | 2015 | INT: Wound | - | VAN S / DAP S / LNZ S |
| 77 | 1272234 | *S. aureus,* MRSA | Czech Republic | 2015 | INT: Wound | - | VAN S / DAP S / LNZ S |
| 78 | 1479327 | *S. aureus,* MRSA | Belgium | 2016 | INT: Abscess | - | VAN S / DAP S / LNZ S |

| # | Organism No | Organism | Country | Year Collected | Body Location | Expected Resistance | Final Resistance |
|---|---|---|---|---|---|---|---|
| 79 | 1480924 | *S. aureus,* MSSA | Netherlands | 2016 | INT: Other | - | VAN S / DAP S / LNZ S |
| 80 | 1480926 | *S. aureus,* MSSA | Netherlands | 2016 | INT: Wound | - | VAN S / DAP S / LNZ S |
| 81 | 1469091 | *S. aureus,* MRSA | France | 2016 | Respiratory: Sputum | LNZ R | VAN S / DAP S / LNZ R |
| 82 | 1319253 | *S. aureus,* MSSA | Belgium | 2015 | INT: Wound | - | VAN S / DAP S / LNZ S |
| 83 | 1311254 | *S. aureus,* MSSA | Denmark | 2015 | INT: Wound | - | VAN S / DAP S / LNZ S |
| 84 | 1332641 | *S. aureus,* MSSA | United States | 2015 | INT: Wound | - | VAN S / DAP S / LNZ S |
| 85 | 1315814 | *S. aureus,* MSSA | Germany | 2015 | INT: Wound | - | VAN S / DAP S / LNZ S |
| 86 | 1228010 | *S. aureus,* MSSA | Canada | 2015 | Bodily Fluids: Abscess/Pus | - | VAN S / DAP S / LNZ S |
| 87 | 1331930 | *S. aureus,* MSSA | United Kingdom | 2015 | INT: Wound | - | VAN S / DAP S / LNZ S |
| 88 | 1174479 | *S. aureus,* MSSA | Croatia | 2015 | INT: Wound | - | VAN S / DAP S / LNZ S |
| 89 | 1322231 | *S. aureus,* MSSA | Spain | 2015 | INT: Wound | - | VAN S / DAP S / LNZ S |
| 90 | 1258353 | *S. aureus,* MRSA | Spain | 2015 | INT: Skin Ulcer | - | VAN S / DAP S / LNZ S |
| 91 | 1262881 | *S. aureus,* MSSA | Germany | 2015 | INT: Abscess | - | VAN S / DAP S / LNZ S |
| 92 | 1283184 | *S. aureus,* MSSA | Turkey | 2015 | INT: Abscess | - | VAN S / DAP S / LNZ S |
| 93 | 1296195 | *S. aureus,* MSSA | Spain | 2015 | INT: Skin | - | VAN S / DAP S / LNZ S |
| 94 | 1280864 | *S. aureus,* MSSA | Portugal | 2015 | INT: Skin Ulcer | - | VAN S / DAP S / LNZ S |
| 95 | 1449510 | *S. aureus,* MSSA | United States | 2016 | INT: Wound | - | VAN S / DAP S / LNZ S |
| 96 | 1464473 | *S. aureus,* MSSA | Canada | 2016 | INT: Wound | - | VAN S / DAP S / LNZ S |
| 97 | 1427879 | *S. aureus,* MSSA | United States | 2016 | INT: Other | - | VAN S / DAP S / LNZ S |
| 98 | 1412354 | *S. aureus,* MSSA | Canada | 2016 | INT: Wound | - | VAN S / DAP S / LNZ S |
| 99 | 1428822 | *S. aureus,* MSSA | United States | 2016 | INT: Wound | - | VAN S / DAP S / LNZ S |
| 100 | 1426641 | *S. aureus,* MRSA | United States | 2016 | INT: Wound | - | VAN S / DAP S / LNZ S |
| 101 | 1537751 | *S. aureus,* MSSA | Canada | 2016 | INT: Wound | - | VAN S / DAP S / LNZ S |
| 102 | 1537741 | *S. aureus,* MSSA | Canada | 2016 | INT: Skin | - | VAN S / DAP S / LNZ S |
| 103 | 1535356 | *S. aureus,* MSSA | United States | 2016 | INT: Wound | - | VAN S / DAP S / LNZ S |
| 104 | 1533772 | *E. faecium* | United States | 2016 | Bodily Fluids: Peritoneal | LNZ R / VAN R | LNZ R / VAN R |
| 105 | 1766256 | *E. faecium* | United States | 2017 | GI: Other | VAN R | LNZ S / VAN R |
| 106 | 1765156 | *E. faecium* | Germany | 2017 | GI: Abscess | VAN R | LNZ R / VAN R |
| 107 | 1569172 | *E. faecalis* | Lithuania | 2017 | GI: Gall Bladder | - | LNZ S / VAN S |
| 108 | 1602010 | *E. faecium* | France | 2017 | GU: Urine | - | LNZ S / VAN S |
| 109 | 1602013 | *E. faecium* | France | 2017 | GU: Urine | - | LNZ S / VAN S |
| 110 | 1606748 | *E. faecalis* | Serbia | 2017 | GU: Urine | - | LNZ S / VAN S |
| 111 | 1766601 | *E. faecalis* | United States | 2017 | GU: Urine | VAN R | LNZ S/ VAN R |
| 112 | 1766602 | *E. faecalis* | United States | 2017 | GU: Urine | VAN R | LNZ S/ VAN R |
| 113 | 1262561 | *S. pyogenes* | Portugal | 2015 | INT: Cellulitis/Erysipelas | MACRO R | MACRO R |
| 114 | 1317874 | *S. pyogenes* | United States | 2015 | INT: Abscess | MACRO R | MACRO R |
| 115 | 1301252 | *S. pyogenes* | United States | 2015 | INT: Cellulitis/Erysipelas | MACRO R | MACRO S |
| 116 | 1201162 | *S. pyogenes* | France | 2015 | INT: Skin | - | MACRO S |
| 117 | 1342786 | *S. pyogenes* | United States | 2015 | INT: Wound | MACRO R | MACRO S |
| 118 | 1261497 | *S. pyogenes* | Italy | 2015 | INT: Abscess | - | MACRO S |
| 119 | 1261500 | *S. pyogenes* | Italy | 2015 | INT: Other | MACRO R | MACRO S |
| 120 | 1279407 | *S. pyogenes* | United Kingdom | 2015 | INT: Wound | - | MACRO S |
| 121 | 1328069 | *S. pyogenes* | Germany | 2015 | INT: Wound | - | MACRO S |

| # | Organism No | Organism | Country | Year Collected | Body Location | Expected Resistance | Final Resistance |
|---|---|---|---|---|---|---|---|
| 122 | 1270153 | *S. pyogenes* | Russia | 2015 | INT: Abscess | - | MACRO S |
| 123 | 1262994 | *S. pyogenes* | Germany | 2015 | INT: Wound | MACRO R | MACRO S |
| 124 | 1247617 | *S. pyogenes* | United States | 2015 | INT: Abscess | - | MACRO S |
| 125 | 1267955 | *S. pyogenes* | United States | 2015 | INT: Wound | MACRO R | MACRO S |
| 126 | 1267719 | *S. pyogenes* | United States | 2015 | INT: Wound | - | MACRO S |
| 127 | 1336219 | *S. pyogenes* | United States | 2015 | INT: Wound | - | MACRO S |
| 128 | 1279084 | *S. pyogenes* | United Kingdom | 2015 | INT: Other | MACRO R | MACRO S |
| 129 | 1424074 | *S. pyogenes* | Italy | 2016 | INT: Abscess | - | MACRO S |
| 130 | 1422650 | *S. pyogenes* | Spain | 2016 | INT: Wound | - | MACRO S |
| 131 | 1428657 | *S. pyogenes* | United States | 2016 | INT: Abscess | - | MACRO S |
| 132 | 1465140 | *S. pyogenes* | Germany | 2016 | INT: Skin Ulcer | MACRO R | MACRO S |
| 133 | 1519165 | *S. pyogenes* | Spain | 2016 | INT: Wound | - | MACRO S |
| 134 | 1447632 | *S. pyogenes* | Poland | 2016 | INT: Wound | MACRO R | MACRO S |
| 135 | 1419666 | *S. pyogenes* | Russia | 2016 | INT: Wound | - | MACRO S |
| 136 | 1413766 | *S. pyogenes* | Russia | 2016 | INT: Wound | MACRO R | MACRO R |
| 137 | 1440834 | *S. pyogenes* | Czech Republic | 2016 | INT: Wound | MACRO R | MACRO R |
| 138 | 1565572 | *S. pneumoniae* | United States | 2017 | Respiratory: Sinuses | - | PEN I |
| 139 | 1612737 | *S. pneumoniae* | Germany | 2017 | GU: Urine | - | PEN I |
| 140 | 1661575 | *S. pneumoniae* | Germany | 2017 | GU: Urine | - | PEN S |
| 141 | 1610983 | *S. pneumoniae* | France | 2017 | GI: Liver | - | PEN S |
| 142 | 1590644 | *S. pneumoniae* | United Kingdom | 2017 | Respiratory: Sputum | PEN R | PEN R |
| 143 | 1590532 | *S. pneumoniae* | United Kingdom | 2017 | Respiratory: Sputum | PEN R | PEN R |
| 144 | 1565469 | *S. pneumoniae* | Switzerland | 2017 | Respiratory: Bronchials | - | PEN S |
| 145 | 1565470 | *S. pneumoniae* | Switzerland | 2017 | Respiratory: Bronchials | - | PEN I |
| 146 | 1621647 | *S. pneumoniae* | Italy | 2017 | Bodily Fluids: Peritoneal | - | PEN S |
| 147 | 1635458 | *S. pneumoniae* | Spain | 2017 | Bodily Fluids: Peritoneal | - | PEN S |
| 148 | 1635459 | *S. pneumoniae* | Spain | 2017 | GI: Abscess | - | PEN S |
| 149 | 1591861 | *S. pneumoniae* | Australia | 2017 | GU: Urine | - | PEN I |
| 150 | 1695310 | *S. pneumoniae* | Taiwan | 2017 | GU: Urine | PEN R | PEN R |
| 151 | 1695316 | *S. pneumoniae* | Taiwan | 2017 | Bodily Fluids: Peritoneal | PEN R | PEN R |
| 152 | 1695116 | *S. pneumoniae* | Philippines | 2017 | Respiratory: Sputum | PEN R | PEN R |
| 153 | 1744977 | *S. pneumoniae* | Czech Republic | 2017 | Bodily Fluids: Peritoneal | - | PEN I |
| 154 | 1654013 | *S. pneumoniae* | United States | 2017 | Respiratory: Bronchials | PEN R | PEN R |
| 155 | 1582680 | *S. pneumoniae* | Czech Republic | 2017 | GU: Other | - | PEN S |
| 156 | 1592877 | *S. pneumoniae* | Australia | 2017 | Respiratory: Sputum | PEN R | PEN R |
| 157 | 1592882 | *S. pneumoniae* | Australia | 2017 | Respiratory: Sputum | PEN R | PEN R |
| 158 | 1616271 | *S. pneumoniae* | Taiwan | 2017 | GU: Urine | PEN R | PEN R |
| 159 | 1598607 | *S. pneumoniae* | Philippines | 2017 | Respiratory: Sputum | - | PEN I |
| 160 | 1519981 | *S. pneumoniae* | France | 2017 | GU: Urine | - | PEN I |
| 161 | 1532565 | *S. pneumoniae* | Vietnam | 2017 | Respiratory: Sputum | PEN R | PEN R |
| 162 | 1734753 | *S. pneumoniae* | Japan | 2017 | Respiratory: Sputum | PEN R | PEN R |
| Spare | 1765227 | *E. faecium* | France | 2017 | GI: Gall Bladder | - | LNZ S / VAN S |
| Spare | 1426580 | *S. aureus*, MRSA | United States | 2016 | INT: Abscess | - | VAN S / DAP S / LNZ S |

| # | Organism No | Organism | Country | Year Collected | Body Location | Expected Resistance | Final Resistance |
|---|---|---|---|---|---|---|---|
| Spare | 1525771 | *S. aureus,* MSSA | United States | 2016 | Bodily Fluids: Abscess / Pus | - | VAN S / DAP S / LNZ S |
| Spare | 1765036 | *E. faecalis* | Korea, South | 2017 | Bodily Fluids: Peritoneal | - | LNZ S / VAN S |
| Spare | 1426536 | *S. pyogenes* | United States | 2016 | INT: Abscess | MACRO R | MACRO R |

DAP: daptomycin; LNZ: linezolid; MACRO: macrolide; PEN: penicillin; VAN: vancomycin; R: resistant; I: intermediate; NS: non-susceptible; S: susceptible. GU: Genital/urinary. GI: Gastrointestinal, INT: Integumentary, CVS: cardiovascular system

**Table S2**. Line listing of the MIC data for 167 the Gram-positive isolates

| | | MIC (µg/mL) | | | | | | | | | | |
|---|---|---|---|---|---|---|---|---|---|---|---|---|
| **Organism No** | **Organism** | **MUP** | **NANO** | **VAN** | **DAP** | **PEN** | **LNZ** | **FOX** | **ERY** | **TET** | **LVX** | **SXT** |
| 626678 | *S. aureus*, MRSA | 0.25 | 0.5 | 2 | 4 | ≤0.06 | 2 | >4 | >4 | 0.12 | >4 | 0.12 |
| 410696 | *S. aureus*, MRSA | 0.25 | 1 | 2 | 2 | >16 | 1 | >4 | >4 | 0.12 | 4 | ≤0.06 |
| 651970 | *S. aureus*, MRSA | 0.25 | 1 | 1 | 0.5 | 8 | >8 | >4 | 0.5 | 0.5 | >4 | 0.5 |
| 672232 | *S. aureus*, MRSA | 0.12 | 0.5 | >16 | 0.5 | >16 | 2 | >4 | >4 | 16 | >4 | ≤0.06 |
| 1146992 | *E. faecium* | 1 | 4 | >16 | 2 | >16 | >8 | >4 | >4 | 4 | >4 | >2 |
| 1180874 | *S. aureus*, MRSA | 0.25 | 1 | 2 | 0.5 | 16 | >8 | >4 | 0.25 | 0.5 | >4 | ≤0.06 |
| 746091 | *S. aureus*, MRSA | 0.25 | 0.5 | 1 | 0.25 | 16 | 2 | >4 | 0.25 | 0.25 | >4 | ≤0.06 |
| 817763 | *S. aureus*, MRSA | 32 | >64 | 2 | 1 | >16 | >8 | >4 | >4 | 4 | >4 | ≤0.06 |
| 651971 | *S. aureus*, MRSA | 0.25 | 1 | 1 | 0.5 | 8 | >8 | >4 | 0.5 | 0.5 | >4 | 0.25 |
| 878234 | *S. aureus*, MRSA | 0.25 | 0.5 | 2 | 4 | 8 | 1 | >4 | 0.25 | 0.12 | >4 | ≤0.06 |
| 649380 | *S. aureus*, MRSA | 0.25 | 0.5 | 4 | 1 | 16 | 1 | >4 | >4 | 0.25 | >4 | ≤0.06 |
| 649390 | *S. aureus*, MRSA | 0.25 | 0.5 | 4 | 1 | >16 | 2 | >4 | >4 | 0.25 | >4 | >2 |
| 649391 | *S. aureus*, MRSA | 16 | >64 | 4 | 1 | >16 | 2 | >4 | >4 | 0.25 | >4 | 0.5 |
| 1308248 | *S. aureus*, MRSA | >64 | >64 | 4 | 2 | >16 | 2 | >4 | >4 | 0.5 | >4 | >2 |
| 1308252 | *S. aureus*, MRSA | 0.25 | 0.5 | 1 | 0.25 | 0.25 | 1 | 1 | 0.25 | 0.25 | 0.12 | 0.25 |
| 1308253 | *S. aureus*, MRSA | >64 | >64 | 2 | 1 | >16 | 1 | >4 | >4 | >32 | >4 | >2 |
| 1308254 | *S. aureus*, MRSA | 0.25 | 0.5 | 4 | 2 | >16 | 1 | >4 | >4 | 32 | >4 | >2 |
| 672230 | *S. aureus*, MRSA | 16 | 32 | >16 | 1 | >16 | 2 | >4 | >4 | 2 | >4 | >2 |
| 672231 | *S. aureus*, MRSA | 0.12 | 0.25 | >16 | 1 | 8 | 1 | >4 | >4 | 4 | >4 | >2 |
| 672233 | *S. aureus*, MRSA | 0.12 | 0.5 | >16 | 0.25 | >16 | 2 | >4 | >4 | 32 | >4 | ≤0.06 |
| 672234 | *S. aureus*, MRSA | 0.25 | 1 | >16 | 0.5 | >16 | 4 | >4 | >4 | 0.5 | >4 | ≤0.06 |
| 672235 | *S. aureus*, MRSA | 0.5 | 0.5 | >16 | 0.5 | 16 | 4 | >4 | >4 | 0.5 | >4 | ≤0.06 |
| 1308256 | *S. aureus*, MRSA | 0.25 | 0.5 | >16 | 0.5 | >16 | 2 | >4 | >4 | 0.25 | >4 | ≤0.06 |
| 1308258 | *S. aureus*, MRSA | 8 | 16 | >16 | 0.5 | 4 | 2 | >4 | >4 | 0.12 | >4 | 0.25 |
| 649401 | *S. aureus*, MRSA | 0.12 | 0.25 | 2 | 1 | >16 | >8 | >4 | 0.25 | 32 | >4 | >2 |
| 649402 | *S. aureus*, MRSA | 0.12 | 0.12 | 2 | 1 | >16 | >8 | >4 | 0.5 | 32 | >4 | 0.25 |
| 649404 | *S. aureus*, MRSA | 16 | 32 | 2 | 0.5 | 16 | 8 | >4 | >4 | 16 | >4 | ≤0.06 |
| 309191 | *S. aureus*, MRSA | 0.25 | 0.5 | 1 | 0.5 | 4 | 4 | >4 | >4 | 0.25 | >4 | 0.12 |
| 114489 | *S. aureus*, MRSA | 0.25 | 0.5 | 1 | 1 | >16 | 2 | >4 | >4 | 0.25 | >4 | 0.12 |
| 860769 | *E. faecalis* | 32 | >64 | 1 | 2 | 2 | 2 | >4 | >4 | 32 | 1 | >2 |
| 862935 | *E. faecalis* | 64 | >64 | 1 | 1 | 2 | 2 | >4 | 0.25 | >32 | 1 | ≤0.06 |
| 925016 | *S. aureus*, MRSA | 0.12 | 0.5 | 1 | 0.5 | 2 | 2 | 1 | 0.25 | 0.5 | 0.25 | ≤0.06 |
| 943616 | *S. aureus*, MRSA | 0.12 | 0.12 | 2 | 4 | >16 | 2 | >4 | 0.25 | 0.25 | >4 | ≤0.06 |
| 1018136 | *S. aureus*, MRSA | 0.25 | 0.25 | 2 | 2 | 16 | 2 | >4 | 0.25 | 0.25 | 4 | ≤0.06 |
| 1018140 | *S. aureus*, MRSA | 0.25 | 0.5 | 2 | 4 | 8 | 2 | >4 | 0.25 | 0.25 | >4 | ≤0.06 |
| 927059 | *S. aureus*, MRSA | 0.5 | 1 | 2 | 4 | >16 | 2 | >4 | >4 | >32 | >4 | 0.25 |
| 927060 | *S. aureus*, MRSA | 0.25 | 1 | 2 | 2 | >16 | 2 | >4 | >4 | >32 | >4 | 0.25 |
| 927061 | *S. aureus*, MRSA | 0.25 | 1 | 2 | 4 | >16 | 2 | >4 | >4 | >32 | >4 | 0.25 |
| 928778 | *S. aureus*, MRSA | >64 | >64 | 1 | 0.25 | >16 | >8 | >4 | 0.25 | 0.5 | >4 | ≤0.06 |
| 1246376 | *S. aureus*, MSSA | 0.12 | 0.5 | 1 | 0.5 | 4 | 2 | 4 | 0.12 | 0.25 | 0.25 | ≤0.06 |
| 1268522 | *S. aureus*, MSSA | 0.25 | 0.5 | 1 | 0.5 | ≤0.06 | 1 | 4 | 0.25 | 0.25 | 0.12 | ≤0.06 |
| 1268530 | *S. aureus*, MRSA | 0.25 | 0.5 | 1 | 0.5 | >16 | 2 | >4 | >4 | 0.25 | >4 | ≤0.06 |
| 1268531 | *S. aureus*, MRSA | 32 | 64 | 1 | 0.5 | >16 | 2 | >4 | >4 | 0.25 | >4 | ≤0.06 |

| | | | | | | | | | | | | |
|---|---|---|---|---|---|---|---|---|---|---|---|---|
| 1268648 | *S. aureus*, MRSA | 0.25 | 0.5 | 1 | 0.5 | 16 | 2 | >4 | >4 | 0.25 | >4 | ≤0.06 |
| 1272493 | *S. aureus*, MSSA | 0.25 | 0.5 | 1 | 0.25 | 0.5 | 2 | 4 | 0.25 | 0.25 | 0.25 | ≤0.06 |
| 1282516 | *S. aureus*, MSSA | 0.25 | 0.5 | 1 | 0.25 | 16 | 2 | 2 | 0.25 | 0.25 | 0.25 | ≤0.06 |
| 1276929 | *S. aureus*, MSSA | 0.12 | 0.5 | 1 | 0.5 | ≤0.06 | 1 | 2 | 0.25 | 0.25 | 0.25 | ≤0.06 |
| 1335968 | *S. aureus*, MSSA | 0.12 | 0.5 | 1 | 0.5 | 0.5 | 2 | 2 | >4 | 0.5 | 0.25 | ≤0.06 |
| 1285320 | *S. aureus*, MRSA | 0.12 | 0.12 | 0.5 | 2 | 0.12 | 0.5 | >4 | ≤0.015 | 0.12 | >4 | 0.25 |
| 1259571 | *S. aureus*, MRSA | 0.25 | 0.5 | 1 | 0.5 | 16 | 2 | >4 | 0.5 | 0.25 | 0.25 | ≤0.06 |
| 1336167 | *S. aureus*, MSSA | 0.12 | 0.5 | 1 | 0.5 | 0.25 | 2 | 4 | 0.25 | 0.25 | 0.25 | ≤0.06 |
| 1267763 | *S. aureus*, MSSA | 0.25 | 0.5 | 1 | 0.5 | ≤0.06 | 2 | 4 | 0.25 | 0.25 | 0.25 | ≤0.06 |
| 1435278 | *S. aureus*, MSSA | 0.25 | 0.5 | 1 | 0.25 | 16 | 2 | 4 | >4 | 0.5 | 0.12 | ≤0.06 |
| 1428168 | *S. aureus*, MSSA | >64 | >64 | 1 | 0.5 | 0.12 | 2 | 4 | 0.25 | 0.25 | 0.25 | ≤0.06 |
| 1475091 | *S. aureus*, MSSA | 0.25 | 0.5 | 1 | 0.5 | 16 | 2 | 4 | 0.25 | 0.25 | >4 | ≤0.06 |
| 1475093 | *S. aureus*, MSSA | 0.25 | 0.5 | 1 | 0.5 | ≤0.06 | 2 | 4 | >4 | 0.5 | 0.25 | ≤0.06 |
| 1518939 | *S. aureus*, MSSA | 0.25 | 0.5 | 1 | 0.5 | 0.12 | 2 | 4 | >4 | 0.12 | 2 | ≤0.06 |
| 1518943 | *S. aureus*, MSSA | 0.25 | 0.25 | 2 | 0.5 | ≤0.06 | 2 | 4 | 0.25 | 0.25 | 0.25 | ≤0.06 |
| 1528968 | *S. aureus*, MRSA | 0.12 | 0.5 | 1 | 0.5 | >16 | 2 | >4 | 0.12 | >32 | >4 | 0.5 |
| 1481133 | *S. aureus*, MRSA | 0.25 | 0.5 | 1 | 0.5 | >16 | 4 | >4 | 0.25 | 0.5 | 0.25 | ≤0.06 |
| 1502818 | *S. aureus*, MSSA | 0.25 | 0.5 | 1 | 0.5 | ≤0.06 | 2 | 4 | 0.25 | 0.25 | 0.25 | ≤0.06 |
| 1502830 | *S. aureus*, MSSA | 0.12 | 0.5 | 0.5 | 0.25 | >16 | 2 | 4 | >4 | 0.25 | 0.12 | ≤0.06 |
| 1502834 | *S. aureus*, MSSA | 0.25 | 0.5 | 1 | 0.5 | 2 | 2 | 4 | 0.25 | 0.25 | 0.25 | ≤0.06 |
| 1540234 | *S. aureus*, MSSA | 0.12 | 0.25 | 1 | 0.25 | 2 | 2 | 2 | 0.25 | 0.12 | 0.12 | 0.12 |
| 1540236 | *S. aureus*, MSSA | 0.25 | 0.5 | 1 | 0.5 | ≤0.06 | 2 | 4 | 0.25 | 0.25 | 0.25 | ≤0.06 |
| 1540244 | *S. aureus*, MSSA | 0.25 | 0.5 | 1 | 1 | ≤0.06 | 2 | 4 | 0.25 | 0.25 | >4 | ≤0.06 |
| 1503418 | *S. aureus*, MSSA | 0.25 | 0.5 | 1 | 0.5 | >16 | 2 | 2 | 0.25 | 0.25 | 0.25 | 0.12 |
| 1503430 | *S. aureus*, MSSA | 0.25 | 0.5 | 1 | 0.5 | ≤0.06 | 2 | 2 | 0.25 | 0.25 | 0.12 | ≤0.06 |
| 1503437 | *S. aureus*, MSSA | 0.25 | 0.5 | 1 | 0.5 | ≤0.06 | 2 | 4 | 2 | 0.25 | 0.25 | ≤0.06 |
| 1503428 | *S. aureus*, MSSA | 0.25 | 0.5 | 0.5 | 0.5 | ≤0.06 | 2 | 2 | 0.25 | 0.25 | 0.25 | ≤0.06 |
| 1479809 | *S. aureus*, MRSA | 0.12 | 0.25 | 1 | 0.5 | >16 | 2 | >4 | >4 | 32 | 4 | ≤0.06 |
| 1551440 | *S. aureus*, MSSA | 0.12 | 0.25 | 1 | 0.5 | 0.5 | 2 | 4 | 0.25 | 0.25 | 0.25 | 0.12 |
| 1284277 | *S. aureus*, MSSA | 0.12 | 0.25 | 1 | 0.5 | ≤0.06 | 2 | 4 | 0.25 | 0.25 | 0.12 | ≤0.06 |
| 1247465 | *S. aureus*, MSSA | 0.25 | 0.25 | 1 | 0.5 | 16 | 2 | 4 | >4 | 0.25 | 0.25 | ≤0.06 |
| 1303514 | *S. aureus*, MRSA | 0.25 | 0.5 | 1 | 0.5 | >16 | 2 | >4 | >4 | 0.25 | >4 | ≤0.06 |
| 1260284 | *S. aureus*, MSSA | 0.25 | 0.5 | 1 | 0.5 | 8 | 2 | 4 | 0.25 | 0.25 | 0.25 | ≤0.06 |
| 1272234 | *S. aureus*, MRSA | 0.12 | 0.25 | 1 | 0.5 | 16 | 2 | >4 | >4 | 0.25 | >4 | 0.5 |
| 1479327 | *S. aureus*, MRSA | 0.12 | 0.25 | 2 | 1 | >16 | 1 | >4 | >4 | >32 | >4 | 0.5 |
| 1480924 | *S. aureus*, MSSA | 0.25 | 1 | 1 | 0.5 | >16 | 2 | 4 | 0.25 | 0.5 | 0.25 | ≤0.06 |
| 1480926 | *S. aureus*, MSSA | 0.25 | 0.5 | 1 | 0.25 | 8 | 2 | 2 | 0.5 | 0.25 | 0.12 | ≤0.06 |
| 1469091 | *S. aureus*, MRSA | 0.12 | 0.5 | 2 | 1 | 16 | >8 | >4 | >4 | >32 | >4 | 0.12 |
| 1319253 | *S. aureus*, MSSA | 0.12 | 0.25 | 1 | 0.5 | 0.12 | 2 | 4 | 0.25 | 0.25 | 0.25 | ≤0.06 |
| 1311254 | *S. aureus*, MSSA | 0.25 | 0.25 | 1 | 0.5 | ≤0.06 | 2 | 4 | 0.12 | 0.25 | 0.12 | ≤0.06 |
| 1332641 | *S. aureus*, MSSA | 0.25 | 0.25 | 1 | 0.5 | ≤0.06 | 2 | 2 | 0.25 | 0.5 | 0.25 | ≤0.06 |
| 1315814 | *S. aureus*, MSSA | 0.25 | 0.5 | 2 | 0.5 | 8 | 4 | 2 | 0.5 | 0.5 | 0.12 | ≤0.06 |
| 1228010 | *S. aureus*, MSSA | 0.25 | 0.5 | 1 | 0.25 | 0.5 | 2 | 2 | 0.5 | 0.25 | 0.12 | ≤0.06 |
| 1331930 | *S. aureus*, MSSA | 0.25 | 0.5 | 1 | 0.5 | >16 | 2 | 4 | 0.25 | 0.25 | 0.25 | ≤0.06 |
| 1174479 | *S. aureus*, MSSA | 0.12 | 0.5 | 1 | 0.5 | 16 | 2 | 2 | 0.25 | 0.25 | 0.25 | ≤0.06 |
| 1322231 | *S. aureus*, MSSA | 0.25 | 0.25 | 1 | 0.5 | 8 | 2 | 2 | 0.5 | 0.5 | 0.5 | ≤0.06 |
| 1258353 | *S. aureus*, MRSA | 0.25 | 0.5 | 1 | 0.5 | >16 | 4 | >4 | >4 | 0.5 | >4 | 0.12 |
| 1262881 | *S. aureus*, MSSA | 0.25 | 0.25 | 1 | 0.5 | ≤0.06 | 2 | 2 | 0.5 | 0.25 | 0.25 | ≤0.06 |

| | | | | | | | | | | | | |
|---|---|---|---|---|---|---|---|---|---|---|---|---|
| 1283184 | *S. aureus*, MSSA | 0.25 | 0.25 | 1 | 0.5 | 0.25 | 2 | 4 | 0.5 | 0.25 | 0.12 | ≤0.06 |
| 1296195 | *S. aureus*, MSSA | 0.25 | 0.5 | 1 | 0.5 | 0.12 | 2 | 4 | 0.25 | 0.25 | 0.25 | ≤0.06 |
| 1280864 | *S. aureus*, MSSA | 0.25 | 0.5 | 1 | 0.5 | ≤0.06 | 2 | 4 | >4 | 0.25 | 0.25 | ≤0.06 |
| 1449510 | *S. aureus*, MSSA | 0.12 | 0.25 | 1 | 0.5 | >16 | 2 | 4 | 0.25 | 0.25 | 0.12 | ≤0.06 |
| 1464473 | *S. aureus*, MSSA | 0.25 | 0.5 | 1 | 0.5 | >16 | 2 | 4 | 0.25 | 0.5 | 1 | ≤0.06 |
| 1427879 | *S. aureus*, MSSA | 0.12 | 0.25 | 0.5 | 0.25 | 0.25 | 2 | 4 | >4 | 0.5 | 0.25 | ≤0.06 |
| 1412354 | *S. aureus*, MSSA | 0.12 | 0.12 | 0.5 | 0.25 | 8 | 2 | 2 | 0.25 | 0.5 | 0.12 | 0.12 |
| 1428822 | *S. aureus*, MSSA | 0.25 | 0.5 | 1 | 0.25 | ≤0.06 | 1 | 2 | 0.12 | 0.25 | 0.12 | ≤0.06 |
| 1426641 | *S. aureus*, MRSA | 0.25 | 0.5 | 1 | 0.5 | >16 | 2 | >4 | >4 | 0.25 | 4 | ≤0.06 |
| 1537751 | *S. aureus*, MSSA | 0.25 | 0.5 | 1 | 0.5 | 8 | 2 | 2 | 0.5 | 0.25 | 0.25 | ≤0.06 |
| 1537741 | *S. aureus*, MSSA | 0.25 | 0.5 | 1 | 0.5 | ≤0.06 | 2 | 4 | 0.25 | 0.25 | 0.5 | ≤0.06 |
| 1535356 | *S. aureus*, MSSA | 0.25 | 0.5 | 1 | 0.5 | ≤0.06 | 2 | 4 | 0.25 | 0.25 | 0.25 | ≤0.06 |
| 1533772 | *E. faecium* | 0.5 | 2 | >16 | 4 | >16 | >8 | >4 | 2 | 32 | >4 | >2 |
| 1766256 | *E. faecium* | 1 | 2 | >16 | 4 | >16 | 4 | >4 | >4 | 16 | >4 | >2 |
| 1765156 | *E. faecium* | 1 | 2 | >16 | 4 | >16 | 8 | >4 | >4 | 0.5 | >4 | >2 |
| 1569172 | *E. faecalis* | >64 | >64 | 1 | 1 | 2 | 2 | >4 | 2 | >32 | 1 | ≤0.06 |
| 1602010 | *E. faecium* | 1 | 2 | 1 | 4 | >16 | 2 | >4 | 2 | >32 | >4 | >2 |
| 1602013 | *E. faecium* | 1 | 2 | 1 | 4 | >16 | 2 | >4 | 2 | >32 | >4 | >2 |
| 1606748 | *E. faecalis* | 32 | >64 | 1 | 2 | 2 | 2 | >4 | 0.25 | 16 | 2 | ≤0.06 |
| 1766601 | *E. faecalis* | 64 | >64 | >16 | 0.5 | 2 | 2 | >4 | >4 | >32 | >4 | >2 |
| 1766602 | *E. faecalis* | 64 | >64 | >16 | 2 | 8 | 2 | >4 | >4 | >32 | >4 | >2 |
| 1262561 | *S. pyogenes* | 0.25 | 0.5 | 0.5 | 0.12 | 0.12 | 2 | 2 | 4 | 0.25 | 0.5 | 0.12 |
| 1317874 | *S. pyogenes* | 0.12 | 0.12 | 0.5 | 0.06 | 0.12 | 1 | 2 | >4 | 0.25 | 0.5 | 0.25 |
| 1301252 | *S. pyogenes* | 0.25 | 0.25 | 0.5 | 0.12 | ≤0.06 | 1 | ≤1 | 0.03 | 0.25 | 0.25 | 0.12 |
| 1201162 | *S. pyogenes* | 0.12 | 0.12 | 0.5 | 0.06 | 0.12 | 1 | ≤1 | 0.03 | 0.25 | 0.5 | 0.12 |
| 1342786 | *S. pyogenes* | 0.25 | 0.12 | 0.5 | 0.12 | ≤0.06 | 1 | ≤1 | 0.03 | 0.25 | 0.5 | ≤0.06 |
| 1261497 | *S. pyogenes* | 0.12 | 0.25 | 0.5 | 0.06 | ≤0.06 | 2 | ≤1 | 0.06 | 0.25 | 0.5 | ≤0.06 |
| 1261500 | *S. pyogenes* | 0.25 | 0.12 | 0.5 | 0.06 | ≤0.06 | 1 | ≤1 | 0.06 | 0.25 | 0.5 | ≤0.06 |
| 1279407 | *S. pyogenes* | 0.12 | 0.25 | 0.5 | 0.06 | 0.12 | 2 | ≤1 | 0.03 | 0.25 | 0.5 | 0.5 |
| 1328069 | *S. pyogenes* | 0.12 | 0.12 | 0.25 | 0.06 | ≤0.06 | 1 | 2 | 0.03 | 32 | 0.5 | 0.25 |
| 1270153 | *S. pyogenes* | 0.25 | 0.25 | 0.5 | 0.06 | ≤0.06 | 4 | 2 | 0.03 | 0.5 | 0.5 | 0.25 |
| 1262994 | *S. pyogenes* | 0.25 | 0.12 | 0.5 | 0.12 | ≤0.06 | 2 | ≤1 | 0.06 | 8 | 0.5 | 0.5 |
| 1247617 | *S. pyogenes* | 0.12 | 0.12 | 0.5 | 0.06 | 0.12 | 1 | 2 | 0.06 | 0.25 | 1 | 0.25 |
| 1267955 | *S. pyogenes* | 0.25 | 0.06 | 0.5 | 0.12 | ≤0.06 | 1 | ≤1 | 0.03 | 0.25 | 0.5 | ≤0.06 |
| 1267719 | *S. pyogenes* | 0.25 | 0.25 | 0.5 | 0.06 | 0.12 | 2 | 2 | 0.06 | 0.25 | 0.5 | 0.12 |
| 1336219 | *S. pyogenes* | 0.12 | 0.12 | 0.5 | 0.06 | 0.12 | 1 | 2 | 0.06 | 0.25 | 2 | 0.12 |
| 1279084 | *S. pyogenes* | 0.12 | 0.25 | 0.25 | 0.06 | ≤0.06 | 0.5 | 2 | 0.03 | 8 | 0.25 | 0.25 |
| 1424074 | *S. pyogenes* | 1 | 4 | 0.5 | 0.25 | 0.12 | 2 | 2 | 0.06 | 0.5 | 1 | 0.12 |
| 1422650 | *S. pyogenes* | 0.12 | 0.12 | 0.5 | 0.06 | 0.12 | 1 | ≤1 | 0.03 | 0.25 | 0.25 | ≤0.06 |
| 1428657 | *S. pyogenes* | 0.12 | 0.12 | 0.5 | 0.06 | 0.12 | 1 | 2 | 0.03 | 0.25 | 0.5 | 0.5 |
| 1465140 | *S. pyogenes* | 0.12 | 0.12 | 0.5 | 0.06 | 0.12 | 1 | 2 | 0.03 | 32 | 0.5 | ≤0.06 |
| 1519165 | *S. pyogenes* | 0.12 | 0.12 | 0.5 | 0.06 | 0.12 | 1 | ≤1 | 0.03 | >32 | 0.5 | 0.12 |
| 1447632 | *S. pyogenes* | ≤0.06 | 0.06 | 0.5 | 0.12 | ≤0.06 | 0.5 | ≤1 | 0.03 | 0.12 | 2 | 0.25 |
| 1419666 | *S. pyogenes* | 0.12 | 0.12 | 0.25 | 0.12 | 0.12 | 1 | 4 | 0.03 | 0.12 | 0.5 | 0.12 |
| 1413766 | *S. pyogenes* | 0.12 | 0.12 | 0.25 | 0.06 | 0.12 | 1 | 4 | >4 | 0.12 | 0.5 | 0.12 |
| 1440834 | *S. pyogenes* | 0.12 | 0.12 | 0.5 | 0.12 | 0.12 | 1 | 2 | >4 | 32 | 0.25 | ≤0.06 |
| 1565572 | *S. pneumoniae* | 0.5 | 0.5 | 0.25 | 0.12 | 0.12 | 1 | 4 | 4 | 0.25 | 1 | 0.12 |
| 1612737 | *S. pneumoniae* | 0.5 | 1 | 0.25 | 0.12 | 0.12 | 1 | 4 | 0.03 | 0.25 | 1 | 0.25 |

| | | | | | | | | | | | | |
|---|---|---|---|---|---|---|---|---|---|---|---|---|
| 1661575 | *S. pneumoniae* | 0.5 | 1 | 0.5 | 0.5 | ≤0.06 | 2 | 4 | 0.03 | 0.25 | 1 | 0.25 |
| 1610983 | *S. pneumoniae* | 0.5 | 1 | 0.25 | ≤0.03 | ≤0.06 | 1 | 4 | 0.03 | 0.25 | 1 | 0.12 |
| 1590644 | *S. pneumoniae* | 0.25 | 1 | 0.25 | 0.25 | 4 | 1 | >4 | >4 | 16 | 1 | >2 |
| 1590532 | *S. pneumoniae* | 0.5 | 1 | 0.5 | 0.12 | 4 | 1 | >4 | >4 | 16 | 1 | >2 |
| 1565469 | *S. pneumoniae* | 0.5 | 1 | 0.25 | 0.12 | ≤0.06 | 1 | 2 | 0.03 | 0.25 | 1 | 0.25 |
| 1565470 | *S. pneumoniae* | 2 | 8 | 0.25 | 0.12 | 0.12 | 1 | ≤1 | 0.03 | 0.25 | 1 | 0.25 |
| 1621647 | *S. pneumoniae* | 4 | 8 | 0.25 | 0.25 | ≤0.06 | 1 | 2 | >4 | >32 | 0.5 | 0.25 |
| 1635458 | *S. pneumoniae* | 0.25 | 0.5 | 0.25 | 0.25 | ≤0.06 | 2 | 2 | 0.06 | 0.25 | 2 | 0.25 |
| 1635459 | *S. pneumoniae* | 4 | 8 | 0.25 | 0.25 | ≤0.06 | 1 | 2 | 0.06 | 0.25 | 1 | 0.25 |
| 1591861 | *S. pneumoniae* | 0.5 | 2 | 0.5 | 0.25 | 0.25 | 1 | 4 | 0.06 | 0.25 | 1 | 0.12 |
| 1695310 | *S. pneumoniae* | 0.25 | 0.5 | 0.5 | 0.25 | 4 | 1 | >4 | >4 | 32 | 1 | >2 |
| 1695316 | *S. pneumoniae* | 0.25 | 0.5 | 1 | 0.25 | 4 | 2 | >4 | >4 | >32 | >4 | >2 |
| 1695116 | *S. pneumoniae* | 2 | 4 | 0.5 | 0.25 | 8 | 1 | >4 | >4 | 32 | 0.5 | 0.25 |
| 1744977 | *S. pneumoniae* | 0.25 | 0.25 | 0.25 | 0.12 | 0.12 | 0.5 | 2 | 0.03 | 0.12 | 1 | ≤0.06 |
| 1654013 | *S. pneumoniae* | 0.5 | 1 | 0.5 | 0.5 | 2 | 1 | >4 | >4 | >32 | 2 | 2 |
| 1582680 | *S. pneumoniae* | 0.25 | 0.25 | 0.25 | 0.25 | ≤0.06 | 1 | ≤1 | 0.03 | 0.25 | 1 | 0.12 |
| 1592877 | *S. pneumoniae* | 2 | 4 | 0.25 | 0.25 | 4 | 1 | >4 | >4 | 32 | 1 | >2 |
| 1592882 | *S. pneumoniae* | 2 | 8 | 0.5 | 0.25 | 4 | 1 | >4 | >4 | 32 | 0.5 | 0.5 |
| 1616271 | *S. pneumoniae* | 4 | 8 | 0.25 | 0.12 | 4 | 1 | >4 | >4 | 32 | 1 | >2 |
| 1598607 | *S. pneumoniae* | 0.25 | 0.25 | 0.25 | 0.25 | 0.12 | 1 | 4 | 0.03 | 32 | 1 | 0.25 |
| 1519981 | *S. pneumoniae* | 1 | 0.5 | 0.5 | 0.5 | 0.12 | 2 | 4 | 0.03 | >32 | 1 | 0.25 |
| 1532565 | *S. pneumoniae* | 4 | 8 | 0.5 | 0.25 | 4 | 1 | >4 | >4 | 32 | 0.5 | >2 |
| 1734753 | *S. pneumoniae* | 1 | 4 | 0.5 | 0.25 | 4 | 1 | >4 | >4 | >32 | >4 | 0.5 |
| 1765227 | *E. faecium* | 0.5 | 1 | 2 | 0.5 | 0.12 | 2 | >4 | >4 | >32 | 2 | ≤0.06 |
| 1426580 | *S. aureus*, MRSA | 0.12 | 0.5 | 1 | 0.5 | >16 | 2 | >4 | >4 | 0.25 | >4 | 0.5 |
| 1525771 | *S. aureus*, MSSA | 0.12 | 0.5 | 1 | 0.5 | ≤0.06 | 4 | 2 | 0.5 | 0.5 | 0.25 | ≤0.06 |
| 1765036 | *E. faecalis* | 64 | >64 | 0.5 | 1 | 2 | 1 | >4 | 0.5 | 0.5 | 1 | ≤0.06 |
| 1426536 | *S. pyogenes* | 0.12 | 0.12 | 0.5 | 0.06 | 0.12 | 1 | ≤1 | >4 | 16 | 0.5 | 0.25 |

MUP: mupirocin; Nano: nano-mupirocin; VAN: vancomycin; DAP: daptomycin; PEN: penicillin; LNZ: linezolid; FOX: cefoxitin; ERY: erythromycin; TER: tetracycline; LVX: levofloxacin; SXT: trimethoprim/sulfametoxazole

**Table S3.** Mupirocin and Nano-mupirocin MICs for MRSA isolates resistant to one or more of vancomycin, daptomycin or linezolid

| | MIC (µg/mL) | | | | |
|---|---|---|---|---|---|
| **Organism No** | **Mupirocin** | **Nano-mupirocin** | **Vancomycin** | **Daptomycin** | **Linezolid** |
| **Vancomycin resistant** | | | | | |
| 672232 | 0.12 | 0.5 | >16 | 0.5 | 2 |
| 672230 | 16 | 32 | >16 | 1 | 2 |
| 672231 | 0.12 | 0.25 | >16 | 1 | 1 |
| 672233 | 0.12 | 0.5 | >16 | 0.25 | 2 |
| 672234 | 0.25 | 1 | >16 | 0.5 | 4 |
| 672235 | 0.5 | 0.5 | >16 | 0.5 | 4 |
| 1308256 | 0.25 | 0.5 | >16 | 0.5 | 2 |
| 1308258 | 8 | 16 | >16 | 0.5 | 2 |
| **Daptomycin non-susceptible** | | | | | |
| 626678 | 0.25 | 0.5 | 2 | 4 | 2 |
| 410696 | 0.25 | 1 | 2 | 2 | 1 |
| 878234 | 0.25 | 0.5 | 2 | 4 | 1 |
| 943616 | 0.12 | 0.12 | 2 | 4 | 2 |
| 1018136 | 0.25 | 0.25 | 2 | 2 | 2 |
| 1018140 | 0.25 | 0.5 | 2 | 4 | 2 |
| 927059 | 0.5 | 1 | 2 | 4 | 2 |
| 927060 | 0.25 | 1 | 2 | 2 | 2 |
| 927061 | 0.25 | 1 | 2 | 4 | 2 |
| 1285320 | 0.12 | 0.12 | 0.5 | 2 | 0.5 |
| 1308248 | >64 | >64 | 4 | 2 | 2 |
| 1308254 | 0.25 | 0.5 | 4 | 2 | 1 |
| **Linezolid resistant** | | | | | |
| 651970 | 0.25 | 1 | 1 | 0.5 | >8 |
| 1180874 | 0.25 | 1 | 2 | 0.5 | >8 |
| 817763 | 32 | >64 | 2 | 1 | >8 |
| 651971 | 0.25 | 1 | 1 | 0.5 | >8 |
| 649401 | 0.12 | 0.25 | 2 | 1 | >8 |
| 649402 | 0.12 | 0.12 | 2 | 1 | >8 |
| 649404 | 16 | 32 | 2 | 0.5 | 8 |
| 928778 | >64 | >64 | 1 | 0.25 | >8 |
| 1469091 | 0.12 | 0.5 | 2 | 1 | >8 |

**Table S4.** MICs of Nano-mupirocin, mupirocin and rifampicin during passage / multi-step selection for nine Gram-positive clinical isolates.

| | | | MIC (µg/mL) over at day | | | | | | | | | | | | | | |
|---|---|---|---|---|---|---|---|---|---|---|---|---|---|---|---|---|---|
| **IHMA number** | **Organism** | **Selecting agent** | **1** | **2** | **3** | **4** | **5** | **6** | **7** | **8** | **9** | **10** | **11** | **12** | **13** | **14** | **15** |
| 649380 | *S. aureus*, MRSA | Nano-mupirocin | 0.5 | 0.5 | 0.5 | 0.5 | 0.5 | 1 | 0.5 | 0.5 | 0.5 | 0.25 | 0.5 | 0.5 | 0.5 | 0.5 | 0.5 |
| | | Mupirocin | 0.25 | 0.25 | 0.25 | 0.25 | 0.25 | 0.25 | 0.25 | 0.25 | 0.25 | 0.25 | 0.25 | 0.25 | 0.25 | 0.25 | 0.25 |
| | | Rifampicin | 256 | 256 | 128 | 256 | 512 | 256 | 512 | 256 | 512 | 512 | >512 | 512 | >512 | 512 | 512 |
| 649390 | *S. aureus*, MRSA | Nano-mupirocin | 1 | 0.5 | 0.5 | 0.5 | 0.5 | 1 | 0.5 | 0.5 | 0.5 | 0.25 | 0.5 | 0.5 | 0.5 | 0.5 | 0.5 |
| | | Mupirocin | 0.25 | 0.25 | 0.5 | 0.5 | 0.25 | 0.25 | 0.25 | 0.25 | 0.25 | 0.25 | 0.25 | 0.25 | 0.25 | 0.5 | 0.25 |
| | | Rifampicin | 0.015 | 0.015 | 0.03 | 0.03 | 0.03 | 0.03 | 0.03 | 0.03 | 0.015 | 0.015 | 0.015 | 0.015 | 0.015 | 0.015 | 0.015 |
| 1308254 | *S. aureus*, MRSA | Nano-mupirocin | 1 | 0.5 | 0.5 | 1 | 0.5 | 1 | 0.5 | 0.5 | 0.5 | 0.25 | 0.5 | 0.5 | 0.5 | 1 | 0.5 |
| | | Mupirocin | 0.25 | 0.25 | 0.25 | 0.25 | 0.25 | 0.25 | 0.25 | 0.25 | 0.25 | 0.25 | 0.25 | 0.25 | 0.25 | 0.25 | 0.25 |
| | | Rifampicin | 2 | 1 | 2 | 2 | 2 | 2 | 2 | 2 | 2 | 2 | 4 | 2 | 2 | 4 | 2 |
| 672231 | *S. aureus*, MRSA | Nano-mupirocin | 0.25 | 0.25 | 0.25 | 0.5 | 0.25 | 0.5 | 0.5 | 0.25 | 0.25 | 0.25 | 0.5 | 0.5 | 0.5 | 1 | 1 |
| | | Mupirocin | 0.12 | 0.12 | 0.25 | 1 | 0.5 | 1 | 0.5 | 0.5 | 1 | 1 | 1 | 1 | 0.5 | 1 | 1 |
| | | Rifampicin | 0.004 | 0.004 | 0.004 | 0.004 | 0.004 | 0.004 | 0.004 | 0.004 | 0.004 | 0.004 | 0.004 | 0.004 | 0.004 | 0.004 | 0.004 |
| 672233 | *S. aureus*, MRSA | Nano-mupirocin | 0.5 | 0.25 | 0.25 | 0.25 | 0.5 | 0.5 | 0.5 | 0.25 | 0.25 | 0.12 | 0.5 | 0.5 | 0.5 | 0.5 | 0.5 |
| | | Mupirocin | 0.12 | 0.12 | 0.12 | 0.12 | 0.25 | 0.12 | 0.12 | 0.12 | 0.25 | 0.25 | 0.25 | 0.25 | 0.25 | 0.25 | 0.5 |
| | | Rifampicin | 0.004 | 0.008 | 0.015 | 0.008 | 0.015 | 0.015 | 0.015 | 0.015 | 0.015 | 0.015 | 0.008 | 0.008 | 0.015 | 0.015 | 0.015 |
| 672232 | *S. aureus*, MRSA | Nano-mupirocin | 0.25 | 0.25 | 0.5 | >32 | 0.5 | 0.5 | 0.5 | 0.5 | 0.5 | 0.25 | 0.5 | 0.5 | 0.5 | 0.5 | 0.5 |
| | | Mupirocin | 0.12 | 0.12 | 0.12 | 0.12 | 0.12 | 0.12 | 0.12 | 0.12 | 0.12 | 0.25 | 0.25 | 0.25 | 0.12 | 0.25 | 0.12 |
| | | Rifampicin | 0.004 | 0.015 | 0.015 | 0.015 | 0.015 | 0.015 | 0.015 | 0.015 | 0.015 | 0.015 | 0.008 | 0.008 | 0.015 | 0.015 | 0.015 |
| 1146992 | *E. faecium* | Nano-mupirocin | 2 | 4 | 4 | 4 | 4 | 4 | 8 | 8 | 8 | 4 | 8 | 8 | 8 | 8 | 8 |
| | | Mupirocin | 1 | 1 | 2 | 2 | 2 | 2 | 2 | 4 | 2 | 4 | 4 | 4 | 4 | 4 | 4 |
| | | Rifampicin | >512 | >512 | >512 | >512 | >512 | >512 | >512 | >512 | >512 | >512 | >512 | >512 | >512 | >512 | >512 |
| 1533772 | *E. faecium* | Nano-mupirocin | 2 | 2 | 4 | 8 | 8 | 16 | 16 | 16 | 16 | 8 | 32 | 16 | 32 | 32 | 32 |
| | | Mupirocin | 0.5 | 0.5 | 0.5 | 1 | 2 | 2 | 2 | 4 | 2 | 4 | 4 | 8 | 4 | 8 | 4 |
| | | Rifampicin | 4 | 8 | 8 | 8 | 16 | 8 | 16 | 16 | 16 | 32 | 16 | 16 | 32 | 16 | 16 |
| 1765156 | *E. faecium* | Nano-mupirocin | 1 | 2 | 1 | 2 | 4 | 4 | 8 | 4 | 4 | 2 | 8 | 16 | 8 | 8 | 8 |
| | | Mupirocin | 0.5 | 1 | 2 | 2 | 2 | 4 | 2 | 2 | 4 | 4 | 8 | 8 | 8 | 8 | 8 |
| | | Rifampicin | >512 | >512 | >512 | >512 | >512 | >512 | >512 | >512 | >512 | >512 | >512 | >512 | >512 | >512 | >512 |

Highlight in red: possible issue with MIC plate on that day

**Table S5.** MICs for potential mutants obtained in multi-step selection, as re-tested after five days of subculture on drug- free agar.

| IHMA no. | Organism | Selective agent | Day | Drug | Original MIC (µg/mL) | MIC when potential mutant was isolated (µg/mL) | MIC after 5 non selective subculture (µg/mL) | Comment |
|---|---|---|---|---|---|---|---|---|
| 649380 | *S. aureus,* MRSA | Rifampicin | 11 | Nano-mupirocin | 0.5 | - | 0.5 | No increase of Nano-mupirocin MIC observed |
| | | | | Mupirocin | 0.25 | - | 0.25 | No increase of Mupirocin MIC observed |
| | | | | Rifampicin | 256 | >512 | >512 | Mutant confirmed for Rifampicin |
| 672231 | *S. aureus,* MRSA | Mupirocin | 4 | Nano-mupirocin | 0.25 | - | 2 | Parallel increase of Nano-Mupirocin MIC |
| | | | | Mupirocin | 0.12 | 1 | 0.5 | Mutant confirmed for Mupirocin |
| | | | | Rifampicin | 0.004 | - | 0.004 | No increase of Rifampicin MIC observed |
| | | Nano-mupirocin | 14 | Nano-mupirocin | 0.25 | 1 | 2 | Mutant confirmed for Nano-Mupirocin |
| | | | | Mupirocin | 0.12 | - | 0.5 | Parallel increase of Mupirocin MIC |
| | | | | Rifampicin | 0.004 | - | 0.004 | No increase of Rifampicin MIC observed |
| 672233 | *S. aureus,* MRSA | Rifampicin | 3 | Nano-mupirocin | 0.5 | - | 0.5 | No increase of Nano-Mupirocin MIC observed |
| | | | | Mupirocin | 0.12 | - | 0.12 | No increase of Mupirocin MIC observed |
| | | | | Rifampicin | 0.004 | 0.015 | 0.008 | Mutant not confirmed |
| | | Mupirocin | 15 | Nano-mupirocin | 0.5 | - | 1 | No increase of Nano-Mupirocin MIC observed |
| | | | | Mupirocin | 0.12 | 0.5 | 0.25 | Mutant not confirmed |
| | | | | Rifampicin | 0.004 | - | 0.008 | No increase of Rifampicin MIC observed |
| 672232 | *S. aureus,* MRSA | Rifampicin | 2 | Nano-mupirocin | 0.25 | - | 0.5 | No increase of Nano-Mupirocin MIC observed |
| | | | | Mupirocin | 0.12 | - | 0.12 | No increase of Mupirocin MIC observed |
| | | | | Rifampicin | 0.004 | 0.015 | 0.008 | Mutant not confirmed |

| IHMA number | Organism | Selecting agent | Day | Drug | Original MIC (µg/mL) | MIC when potential mutant was isolated (µg/mL) | MIC after 5 non-selective subcultures (µg/mL) | Comment |
|---|---|---|---|---|---|---|---|---|
| 1146992 | *E. faecium* | Nano-mupirocin | 7 | Nano-mupirocin | 2 | 8 | 4 | Mutant not confirmed |
| | | | | Mupirocin | 1 | - | 1 | No increase of Mupirocin MIC observed |
| | | | | Rifampicin | >512 | - | >512 | No increase of Rifampicin MIC observed |
| | | Mupirocin | 8 | Nano-mupirocin | 2 | - | 4 | No increase of Nano-Mupirocin MIC observed |
| | | | | Mupirocin | 1 | 4 | 1 | Mutant not confirmed |
| | | | | Rifampicin | >512 | - | >512 | No increase of rifampicin MIC observed |
| 1533772 | *E. faecium* | Nano-mupirocin | 4 | Nano-mupirocin | 2 | 8 | 8 | Mutant confirmed for nano-mupirocin |
| | | | | Mupirocin | 0.5 | - | 2 | Parallel increase of mupirocin MIC |
| | | | | Rifampicin | 4 | - | 4 | No increase of rifampicin MIC observed |
| | | Mupirocin | 5 | Nano-mupirocin | 2 | - | 8 | Parallel increase of nano-mupirocin MIC |
| | | | | Mupirocin | 0.5 | 2 | 2 | Mutant confirmed for mupirocin |
| | | | | Rifampicin | 4 | - | 4 | No increase of rifampicin MIC observed |
| | | Rifampicin | 5 | Nano-mupirocin | 2 | - | 2 | No increase of nano-mupirocin MIC observed |
| | | | | Mupirocin | 0.5 | - | 0.5 | No increase of mupirocin MIC observed |
| | | | | Rifampicin | 4 | 16 | 8 | Mutant not confirmed |
| | | Nano-mupirocin | 11 | Nano-mupirocin | 2 | 32 | 32 | Mutant confirmed for nano-mupirocin |
| | | | | Mupirocin | 0.5 | - | 8 | Parallel increase of mupirocin MIC |
| | | | | Rifampicin | 4 | - | 2 | No increase of rifampicin MIC observed |
| | | Mupirocin | 12 | Nano-mupirocin | 2 | - | 16 | Parallel increase of nano-mupirocin MIC |
| | | | | Mupirocin | 0.5 | 8 | 4 | Mutant confirmed for mupirocin |
| | | | | Rifampicin | 4 | - | 4 | No increase of rifampicin MIC observed |
| 1765156 | *E. faecium* | Mupirocin | 3 | Nano-mupirocin | 1 | - | 4 | Increase of nano-mupirocin MIC |
| | | | | Mupirocin | 0.5 | 2 | 1 | Mutant not confirmed |
| | | | | Rifampicin | >512 | - | >512 | - |
| | | Nano-mupirocin | 5 | Nano-mupirocin | 1 | 4 | 4 | Mutant confirmed for nano-mupirocin |
| | | | | Mupirocin | 0.5 | - | 1 | No increase of mupirocin MIC observed |
| | | | | Rifampicin | >512 | - | >512 | - |
| | | Mupirocin | 11 | Nano-mupirocin | 1 | - | 32 | Parallel increase of nano-mupirocin MIC |
| | | | | Mupirocin | 0.5 | 8 | 8 | Mutant confirmed for mupirocin |
| | | | | Rifampicin | >512 | - | >512 | - |
| | | Nano-mupirocin | 12 | Nano-mupirocin | 1 | 16 | 8 | Mutant confirmed for nano-mupirocin |
| | | | | Mupirocin | 0.5 | - | 2 | Parallel increase of mupirocin MIC |
| | | | | Rifampicin | >512 | - | >512 | - |

**Table S6.** Line listing of agar MIC data for Nano-mupirocin, mupirocin, and rifampicin against nine Gram-positive clinical isolates, as tested at different inocula, and for two QC strains.

| | | | MIC agar (µg/mL) | | |
|---|---|---|---|---|---|
| **Organism number** | **Organism** | **Inoculum size (CFU/spot)** | **Nano-mupirocin (diffusion)[a]** | **Mupirocin** | **Rifampicin** |
| ATCC 29213 | *S. aureus* | $10^4$ (Standard) | 0.12 | 0.12 | 0.004 |
| ATCC 29213 | *S. aureus* | $10^4$ (Standard) | 0.12 | 0.12 | 0.004 |
| ATCC 29213 | *S. aureus* | $10^4$ (Standard) | 0.12 | 0.06 | 0.004 |
| ATCC 29212 | *E. faecalis* | $10^4$ (Standard) | 32 | 16 | 1 |
| ATCC 29212 | *E. faecalis* | $10^4$ (Standard) | 32 | 32 | 1 |
| ATCC 29212 | *E. faecalis* | $10^4$ (Standard) | 32 | 32 | 1 |
| 649380 | *S. aureus*, MRSA | $10^4$ (Standard) | 0.12 | 0.12 | >512 |
| 649380 | *S. aureus*, MRSA | $10^7$ | **0.25** | **0.25** | **>512** |
| 649380 | *S. aureus*, MRSA | $10^6$ | 0.25 | 0.25 | >512 |
| 649380 | *S. aureus*, MRSA | $10^5$ | 0.12 | 0.25 | >512 |
| 649390 | *S. aureus*, MRSA | $10^4$ (Standard) | 0.25 | 0.25 | 0.015 |
| 649390 | *S. aureus*, MRSA | $10^7$ | **2** | **1** | **0.015** |
| 649390 | *S. aureus*, MRSA | $10^6$ | 0.5 | 0.25 | 0.015 |
| 649390 | *S. aureus*, MRSA | $10^5$ | 0.25 | 0.25 | 0.015 |
| 1308254 | *S. aureus*, MRSA | $10^4$ (Standard) | 0.25 | 0.25 | 1 |
| 1308254 | *S. aureus*, MRSA | $10^7$ | **0.5** | **1** | **2** |
| 1308254 | *S. aureus*, MRSA | $10^6$ | 0.25 | 0.25 | 1 |
| 1308254 | *S. aureus*, MRSA | $10^5$ | 0.25 | 0.25 | 1 |
| 672231 | *S. aureus*, MRSA | $10^4$ (Standard) | 0.12 | 0.12 | ≤0.002 |
| 672231 | *S. aureus*, MRSA | $10^7$ | **0.5** | **0.25** | **≤0.002** |
| 672231 | *S. aureus*, MRSA | $10^6$ | 0.12 | 0.12 | ≤0.002 |
| 672231 | *S. aureus*, MRSA | $10^5$ | 0.12 | 0.06 | ≤0.002 |
| 672233 | *S. aureus*, MRSA | $10^4$ (Standard) | 0.12 | 0.06 | 0.004 |
| 672233 | *S. aureus*, MRSA | $10^7$ | **0.5** | **1** | **0.015** |
| 672233 | *S. aureus*, MRSA | $10^6$ | 0.12 | 0.25 | 0.008 |
| 672233 | *S. aureus*, MRSA | $10^5$ | 0.12 | 0.12 | 0.008 |
| 672232 | *S. aureus*, MRSA | $10^4$ (Standard) | 0.06 | ≤0.015 | 0.004 |
| 672232 | *S. aureus*, MRSA | $10^7$ | **0.5** | **1** | **0.015** |
| 672232 | *S. aureus*, MRSA | $10^6$ | 0.06 | 0.12 | 0.008 |
| 672232 | *S. aureus*, MRSA | $10^5$ | 0.06 | 0.06 | 0.004 |
| 1146992 | *E. faecium* | $10^4$ (Standard) | no growth | no growth | >512 |
| 1146992 | *E. faecium* | $10^7$ | **2** | **4** | **>512** |
| 1146992 | *E. faecium* | $10^6$ | 2 | 2 | >512 |
| 1146992 | *E. faecium* | $10^5$ | 1 | 2 | >512 |
| 1533772 | *E. faecium* | $10^4$ (Standard) | 1 | 1 | 4 |
| 1533772 | *E. faecium* | $10^7$ | **2** | **2** | **16** |
| 1533772 | *E. faecium* | $10^6$ | 1 | 1 | 8 |
| 1533772 | *E. faecium* | $10^5$ | 1 | 1 | 8 |
| 1765156 | *E. faecium* | $10^4$ (Standard) | 1 | 0.5 | >512 |
| 1765156 | *E. faecium* | $10^7$ | **2** | **4** | **>512** |
| 1765156 | *E. faecium* | $10^6$ | 2 | 2 | >512 |
| 1765156 | *E. faecium* | $10^5$ | 1 | 1 | >512 |

In bold: MIC used to calculate the concentrations for the Spontaneous mutation frequency (SMF) experiment.

a- Nano-mupirocin was spread on petri dishes containing solidified supplemented QC agar. The plates were allowed to dry at room temperature before bacteria were added. This method was used to prevent nano-mupirocin liposome degradation at temperature higher than 40°C that are usually required to prepare agar panels

**Table S7**. Line listing of single-step spontaneous mutation frequency data for nine Gram-positive isolates tested with Nano-mupirocin, mupirocin, and rifampicin

| IHMA number | Organism | Inoculum | Selective agent [μg/mL] | Selective agent (× MIC) | Potential mutants at 24 h incubation | Confirmed mutants at 24 h | SMF at 24h |
|---|---|---|---|---|---|---|---|
| 649380 | *S. aureus*, MRSA | 2.52E+09 | Nano-mupirocin [1] | 4 × | 2 | 1/2 | 3.97E-10 |
| 649380 | *S. aureus*, MRSA | 2.52E+09 | Nano-mupirocin [2] | 8 × | 5 | 2/2 | 1.98E-09 |
| 649380 | *S. aureus*, MRSA | 2.52E+09 | Nano-mupirocin [4] | 16 × | 6 | 2/2 | 2.38E-09 |
| 649380 | *S. aureus*, MRSA | 2.52E+09 | Mupirocin [1] | 4 × | 0 | | <3.97E-10 |
| 649380 | *S. aureus*, MRSA | 2.52E+09 | Mupirocin [2] | 8 × | 0 | | <3.97E-10 |
| 649380 | *S. aureus*, MRSA | 2.52E+09 | Mupirocin [4] | 16 × | 0 | | <3.97E-10 |
| 649390 | *S. aureus*, MRSA | 1.92E+09 | Nano-mupirocin [8] | 4 × | 0 | | <5.21E-10 |
| 649390 | *S. aureus*, MRSA | 1.92E+09 | Nano-mupirocin [16] | 8 × | 0 | | <5.21E-10 |
| 649390 | *S. aureus*, MRSA | 1.92E+09 | Nano-mupirocin [32] | 16 × | 0 | | <5.21E-10 |
| 649390 | *S. aureus*, MRSA | 1.92E+09 | Mupirocin [4] | 4 × | 0 | | <5.21E-10 |
| 649390 | *S. aureus*, MRSA | 1.92E+09 | Mupirocin [8] | 8 × | 0 | | <5.21E-10 |
| 649390 | *S. aureus*, MRSA | 1.92E+09 | Mupirocin [16] | 16 × | 0 | | <5.21E-10 |
| 649390 | *S. aureus*, MRSA | 1.92E+09 | Rifampicin [0.06] | 4 × | 50 | 2/2 | 2.60E-08 |
| 649390 | *S. aureus*, MRSA | 1.92E+09 | Rifampicin [0.12] | 8 × | 32 | 2/2 | 1.67E-08 |
| 649390 | *S. aureus*, MRSA | 1.92E+09 | Rifampicin [0.25] | 16 × | 26 | 2/2 | 1.35E-08 |
| 1308254 | *S. aureus*, MRSA | 8.00E+08 | Nano-mupirocin [2] | 4 × | 29 | 2/2 | 3.63E-08 |
| 1308254 | *S. aureus*, MRSA | 8.00E+08 | Nano-mupirocin [4] | 8 × | 27 | 2/2 | 3.38E-08 |
| 1308254 | *S. aureus*, MRSA | 8.00E+08 | Nano-mupirocin [8] | 16 × | 73 | 2/2 | 9.13E-08 |
| 1308254 | *S. aureus*, MRSA | 8.00E+08 | Mupirocin [4] | 4 × | 0 | | <1.25E-09 |
| 1308254 | *S. aureus*, MRSA | 8.00E+08 | Mupirocin [8] | 8 × | 0 | | <1.25E-09 |
| 1308254 | *S. aureus*, MRSA | 8.00E+08 | Mupirocin [16] | 16 × | 0 | | <1.25E-09 |
| 1308254 | *S. aureus*, MRSA | 8.00E+08 | Rifampicin [8] | 4 × | >600 | 2/2 | >7.5E-07 |
| 1308254 | *S. aureus*, MRSA | 8.00E+08 | Rifampicin [16] | 8 × | >600 | 2/2 | >7.5E-07 |
| 1308254 | *S. aureus*, MRSA | 8.00E+08 | Rifampicin [32] | 16 × | >600 | 2/2 | >7.5E-07 |
| 672231 | *S. aureus*, MRSA | 2.06E+09 | Nano-mupirocin [2] | 4 × | 3 | 1/2 | 7.28E-10 |
| 672231 | *S. aureus*, MRSA | 2.06E+09 | Nano-mupirocin [4] | 8 × | 0 | | <4.85E-10 |
| 672231 | *S. aureus*, MRSA | 2.06E+09 | Nano-mupirocin [8] | 16 × | 1 | 1/1 | 4.85E-10 |
| 672231 | *S. aureus*, MRSA | 2.06E+09 | Mupirocin [1] | 4 × | 0 | | <4.85E-10 |
| 672231 | *S. aureus*, MRSA | 2.06E+09 | Mupirocin [2] | 8 × | 0 | | <4.85E-10 |

| IHMA number | Organism | Inoculum | Selective agent [µg/mL] | Selective agent (× MIC) | Potential mutants at 24 h incubation | Confirmed mutants at 24 h | SMF at 24h |
|---|---|---|---|---|---|---|---|
| 672231 | *S. aureus*, MRSA | 2.06E+09 | Mupirocin [4] | 16 × | 0 | | <4.85E-10 |
| 672231 | *S. aureus*, MRSA | 2.06E+09 | Rifampicin [0.008] | 4 × | Lawn | 0/2 | <4.85E-10 |
| 672231 | *S. aureus*, MRSA | 2.06E+09 | Rifampicin [0.015] | 8 × | 41 | 2/2 | 1.99E-08 |
| 672231 | *S. aureus*, MRSA | 2.06E+09 | Rifampicin [0.03] | 16 × | 42 | 2/2 | 2.04E-08 |
| 672233 | *S. aureus*, MRSA | 1.48E+09 | Nano-mupirocin [2] | 4 × | 0 | | <6.76E-10 |
| 672233 | *S. aureus*, MRSA | 1.48E+09 | Nano-mupirocin [4] | 8 × | 0 | | <6.76E-10 |
| 672233 | *S. aureus*, MRSA | 1.48E+09 | Nano-mupirocin [8] | 16 × | 0 | | <6.76E-10 |
| 672233 | *S. aureus*, MRSA | 1.48E+09 | Mupirocin [4] | 4 × | 0 | | <6.76E-10 |
| 672233 | *S. aureus*, MRSA | 1.48E+09 | Mupirocin [8] | 8 × | 0 | | <6.76E-10 |
| 672233 | *S. aureus*, MRSA | 1.48E+09 | Mupirocin [16] | 16 × | 0 | | <6.76E-10 |
| 672233 | *S. aureus*, MRSA | 1.48E+09 | Rifampicin [0.06] | 4 × | 160 | 2/2 | 1.08E-07 |
| 672233 | *S. aureus*, MRSA | 1.48E+09 | Rifampicin [0.12] | 8 × | >349 | 2/2 | >2.36E-07 |
| 672233 | *S. aureus*, MRSA | 1.48E+09 | Rifampicin [0.25] | 16 × | >600 | 2/2 | >4.05E-07 |
| 672232 | *S. aureus*, MRSA | 2.22E+09 | Nano-mupirocin [2] | 4 × | 1 | 1/1 | 4.50E-10 |
| 672232 | *S. aureus*, MRSA | 2.22E+09 | Nano-mupirocin [4] | 8 × | 1 | 1/1 | 4.50E-10 |
| 672232 | *S. aureus*, MRSA | 2.22E+09 | Nano-mupirocin [8] | 16 × | 0 | | <4.50E-10 |
| 672232 | *S. aureus*, MRSA | 2.22E+09 | Mupirocin [4] | 4 × | 0 | | <4.50E-10 |
| 672232 | *S. aureus*, MRSA | 2.22E+09 | Mupirocin [8] | 8 × | 0 | | <4.50E-10 |
| 672232 | *S. aureus*, MRSA | 2.22E+09 | Mupirocin [16] | 16 × | 1 | 0/1 | <4.50E-10 |
| 672232 | *S. aureus*, MRSA | 2.22E+09 | Rifampicin [0.06] | 4 × | 128 | 2/2 | 5.77E-08 |
| 672232 | *S. aureus*, MRSA | 2.22E+09 | Rifampicin [0.12] | 8 × | 79 | 2/2 | 3.56E-08 |
| 672232 | *S. aureus*, MRSA | 2.22E+09 | Rifampicin [0.25] | 16 × | 18 | 2/2 | 8.11E-09 |
| 1146992 | *E. faecium* | 5.55E+08 | Nano-mupirocin [8] | 4 × | 3 | 0/2 | <1.80E-09 |
| 1146992 | *E. faecium* | 5.55E+08 | Nano-mupirocin [16] | 8 × | 12 | 0/2 | <1.80E-09 |
| 1146992 | *E. faecium* | 5.55E+08 | Nano-mupirocin [32] | 16 × | 4 | 1/2 | 3.60E-09 |
| 1146992 | *E. faecium* | 5.55E+08 | Mupirocin [16] | 4 × | 0 | | <1.80E-09 |
| 1146992 | *E. faecium* | 5.55E+08 | Mupirocin [32] | 8 × | 0 | | <1.80E-09 |
| 1146992 | *E. faecium* | 5.55E+08 | Mupirocin [64] | 16 × | 0 | | <1.80E-09 |
| 1533772 | *E. faecium* | 1.36E+09 | Nano-mupirocin [8] | 4 × | 0 | | <7.35E-10 |
| 1533772 | *E. faecium* | 1.36E+09 | Nano-mupirocin [16] | 8 × | 0 | | <7.35E-10 |
| 1533772 | *E. faecium* | 1.36E+09 | Nano-mupirocin [32] | 16 × | 0 | | <7.35E-10 |
| 1533772 | *E. faecium* | 1.36E+09 | Mupirocin [8] | 4 × | 0 | | <7.35E-10 |
| 1533772 | *E. faecium* | 1.36E+09 | Mupirocin [16] | 8 × | 0 | | <7.35E-10 |
| 1533772 | *E. faecium* | 1.36E+09 | Mupirocin [32] | 16 × | 0 | | <7.35E-10 |
| 1533772 | *E. faecium* | 1.36E+09 | Rifampicin [64] | 4 × | 63 | 2/2 | 4.63E-08 |

| IHMA number | Organism | Inoculum | Selective agent [µg/mL] | Selective agent (× MIC) | Potential mutants at 24 h incubation | Confirmed mutants at 24 h | SMF at 24h |
|---|---|---|---|---|---|---|---|
| 1533772 | *E. faecium* | 1.36E+09 | Rifampicin [128] | 8 × | 82 | 2/2 | 6.03E-08 |
| 1533772 | *E. faecium* | 1.36E+09 | Rifampicin [256] | 16 × | 60 | 2/2 | 4.41E-08 |
| 1765156 | *E. faecium* | 1.46E+09 | Nano-mupirocin [8] | 4 × | 2 | 1/2 | 6.85E-10 |
| 1765156 | *E. faecium* | 1.46E+09 | Nano-mupirocin [16] | 8 × | 5 | 2/2 | 3.42E-09 |
| 1765156 | *E. faecium* | 1.46E+09 | Nano-mupirocin [32] | 16 × | 1 | 0/1 | <6.85E-10 |
| 1765156 | *E. faecium* | 1.46E+09 | Mupirocin [16] | 4 × | 0 | | <6.85E-10 |
| 1765156 | *E. faecium* | 1.46E+09 | Mupirocin [32] | 8 × | 0 | | <6.85E-10 |
| 1765156 | *E. faecium* | 1.46E+09 | Mupirocin [64] | 16 × | 1 | 0/1 | <6.85E-10 |

**Table S8.** Line listing of MICs for variants selected in spontaneous mutation frequency experiments.

| Label | MIC (µg/mL) | | | Comment |
|---|---|---|---|---|
| | Nano-mupirocin | Mupirocin | Rifampicin | |
| *S. aureus*, MRSA. 649380 parent isolate | 0.5 | 0.25 | >512 | - |
| 11- 649380- NANO-MUP- 1 µg/mL- 24H.1 | 0.5 | 0.25 | >512 | Not confirmed |
| 11- 649380- NANO-MUP- 1 µg/mL- 24H.2 | 32 | 16 | >512 | Confirmed, increase in MUP MIC |
| 11- 649380- NANO-MUP- 2 µg/mL- 24H.1 | 32 | 16 | >512 | Confirmed, increase in MUP MIC |
| 11- 649380- NANO-MUP- 2 µg/mL- 24H.2 | 64 | 16 | >512 | Confirmed, increase in MUP MIC |
| 11- 649380- NANO-MUP- 4 µg/mL- 24H.1 | 64 | 16 | >512 | Confirmed, increase in MUP MIC |
| 11- 649380- NANO-MUP- 4 µg/mL- 24H.2 | 64 | 16 | >512 | Confirmed, increase in MUP MIC |
| *S. aureus*, MRSA. 649390 parent isolate | 1 | 0.25 | 0.015 | - |
| 12- 649390- RIF- 0.06 µg/mL- 24H.1 | 1 | 0.25 | 16 | Mutant confirmed |
| 12- 649390- RIF- 0.06 µg/mL- 24H.2 | 1 | 0.25 | 128 | Mutant confirmed |
| 12- 649390- RIF- 0.12 µg/mL- 24H.1 | 2 | 0.25 | 16 | Mutant confirmed |
| 12- 649390- RIF- 0.12 µg/mL- 24H.2 | 2 | 0.25 | 256 | Mutant confirmed |
| 12- 649390- RIF- 0.25 µg/mL- 24H.1 | 2 | 0.5 | 256 | Mutant confirmed |
| 12- 649390- RIF- 0.25 µg/mL- 24H.2 | 2 | 0.5 | >512 | Mutant confirmed |
| *S. aureus*, MRSA. 1308254 | 1 | 0.25 | 2 | - |
| 17- 1308254- NANO-MUP- 2 µg/mL- 24H.1 | 64 | 16 | 2 | Confirmed, increase in MUP MIC |
| 17- 1308254- NANO-MUP- 2 µg/mL- 24H.2 | 64 | 16 | 2 | Confirmed, increase in MUP MIC |
| 17- 1308254- NANO-MUP- 4 µg/mL- 24H.1 | 32 | 8 | 2 | Confirmed, increase in MUP MIC |
| 17- 1308254- NANO-MUP- 4 µg/mL- 24H.2 | 32 | 16 | 2 | Confirmed, increase in MUP MIC |
| 17- 1308254- NANO-MUP- 8 µg/mL- 24H.1 | 32 | 8 | 2 | Confirmed, increase in MUP MIC |
| 17- 1308254- NANO-MUP- 8 µg/mL- 24H.2 | 32 | 16 | 2 | Confirmed, increase in MUP MIC |
| 17- 1308254- RIF- 8 µg/mL- 24H.1 | 2 | 0.25 | >512 | Mutant confirmed |
| 17- 1308254- RIF- 8 µg/mL- 24H.2 | 0.5 | 0.25 | >512 | Mutant confirmed |
| 17- 1308254- RIF- 16 µg/mL- 24H.1 | 1 | 0.25 | >512 | Mutant confirmed |
| 17- 1308254- RIF- 16 µg/mL- 24H.2 | 0.5 | 0.25 | >512 | Mutant confirmed |
| 17- 1308254- RIF- 32 µg/mL- 24H.1 | 0.5 | 0.25 | >512 | Mutant confirmed |
| 17- 1308254- RIF- 32 µg/mL- 24H.2 | 0.5 | 0.25 | >512 | Mutant confirmed |
| *S. aureus*, MRSA. 672231 | 0.25 | 0.06 | 0.004 | - |
| 19- 672231- NANO-MUP- 2 µg/mL- 24H.1 | 32 | 8 | 0.004 | Confirmed, increase in MUP MIC |
| 19- 672231- NANO-MUP- 2 µg/mL- 24H.2 | 0.25 | 0.12 | 0.004 | Not confirmed |
| 19- 672231- NANO-MUP- 8 µg/mL- 24H.1 | 32 | 8 | 0.004 | Confirmed, increase in MUP MIC |
| 19- 672231- RIF- Lawn- 24H.1 | 0.25 | 0.12 | 0.004 | Not confirmed |
| 19- 672231- RIF- Lawn- 24H.2 | 0.25 | 0.12 | 0.004 | Not confirmed |
| 19- 672231- RIF- 0.015 µg/mL- 24H.1 | 0.5 | 0.12 | 0.06 | Mutant confirmed |
| 19- 672231- RIF- 0.015 µg/mL- 24H.2 | 0.25 | 0.06 | 1 | Mutant confirmed |
| 19- 672231- RIF- 0.03 µg/mL- 24H.1 | 0.25 | 0.12 | 32 | Mutant confirmed |
| 19- 672231- RIF- 0.03 µg/mL- 24H.2 | 0.25 | 0.12 | 512 | Mutant confirmed |
| *S. aureus*, MRSA. 672233 | 0.5 | 0.06 | 0.008 | - |
| 20- 672233- RIF- 0.06 µg/mL- 24H.1 | 0.5 | 0.12 | 512 | Mutant confirmed |
| 20- 672233- RIF- 0.06 µg/mL- 24H.2 | 0.5 | 0.12 | 256 | Mutant confirmed |
| 20- 672233- RIF- 0.12 µg/mL- 24H.1 | 1 | 0.25 | 1 | Mutant confirmed |
| 20- 672233- RIF- 0.12 µg/mL- 24H.2 | 0.5 | 0.12 | 512 | Mutant confirmed |
| 20- 672233- RIF- 0.25 µg/mL- 24H.1 | 1 | 0.25 | 2 | Mutant confirmed |

| | MIC (µg/mL) | | | |
|---|---|---|---|---|
| **Label** | **Nano-mupirocin** | **Mupirocin** | **Rifampicin** | **Comment** |
| 20- 672233- RIF- 0.25 µg/mL- 24H.2 | 0.5 | 0.12 | 512 | Mutant confirmed |
| *S. aureus*, MRSA. 672232 | 0.5 | 0.06 | 0.004 | - |
| 4- 672232- NANO-MUP- 2 µg/mL- 24H.1 | 64 | 16 | 0.008 | Confirmed, increase in MUP MIC |
| 4- 672232- NANO-MUP- 4 µg/mL- 24H.1 | 4 | 1 | 0.008 | Confirmed, increase in MUP MIC |
| 4- 672232- MUP- 16 µg/mL- 24H.1 | 0.5 | 0.12 | 0.008 | Not confirmed |
| 4- 672232- RIF- 0.06 µg/mL- 24H.1 | 0.5 | 0.12 | 0.5 | Mutant confirmed |
| 4- 672232- RIF- 0.06 µg/mL- 24H.2 | 0.5 | 0.12 | 0.5 | Mutant confirmed |
| 4- 672232- RIF- 0.12 µg/mL- 24H.1 | 0.5 | 0.12 | 2 | Mutant confirmed |
| 4- 672232- RIF- 0.12 µg/mL- 24H.2 | 0.5 | 0.12 | 4 | Mutant confirmed |
| 4- 672232- RIF- 0.25 µg/mL- 24H.1 | 0.5 | 0.12 | >512 | Mutant confirmed |
| 4- 672232- RIF- 0.25 µg/mL- 24H.2 | 0.5 | 0.12 | >512 | Mutant confirmed |
| *E. faecium.* 1146992 | 4 | 1 | >512 | - |
| 5- 1146992- NANO-MUP- 8 µg/mL- 24H.1 | 4 | 1 | >512 | Not confirmed |
| 5- 1146992- NANO-MUP- 8 µg/mL- 24H.2 | 4 | 1 | >512 | Not confirmed |
| 5- 1146992- NANO-MUP- 16 µg/mL- 24H.1 | 4 | 1 | >512 | Not confirmed |
| 5- 1146992- NANO-MUP- 16 µg/mL- 24H.2 | 4 | 1 | >512 | Not confirmed |
| 5- 1146992- NANO-MUP- 32 µg/mL- 24H.1 | 32 | 8 | >512 | Confirmed, increase in MUP MIC |
| 5- 1146992- NANO-MUP- 32 µg/mL- 24H.2 | 8 | 1 | >512 | Not confirmed |
| *E. faecium.* 1533772 | 2 | 0.5 | 4 | - |
| 104- 1533772- RIF- 64 µg/mL- 24H.1 | 2 | 0.5 | >512 | Mutant confirmed |
| 104- 1533772- RIF- 64 µg/mL- 24H.2 | 2 | 0.5 | >512 | Mutant confirmed |
| 104- 1533772- RIF- 128 µg/mL- 24H.1 | 1 | 0.5 | 512 | Mutant confirmed |
| 104- 1533772- RIF- 128 µg/mL- 24H.2 | 2 | 0.5 | 512 | Mutant confirmed |
| 104- 1533772- RIF- 256 µg/mL- 24H.1 | 2 | 0.5 | 512 | Mutant confirmed |
| 104- 1533772- RIF- 256 µg/mL- 24H.2 | 2 | 0.5 | 512 | Mutant confirmed |
| *E. faecium.* 1765156 | 2 | 0.5 | >512 | - |
| 106- 1765156- NANO-MUP- 8 µg/mL- 24H.1 | 16 | 4 | >512 | Confirmed, increase in MUP MIC |
| 106- 1765156- NANO-MUP- 8 µg/mL- 24H.2 | 2 | 1 | >512 | Not confirmed |
| 106- 1765156- NANO-MUP- 16 µg/mL- 24H.1 | 32 | 8 | >512 | Confirmed, increase in MUP MIC |
| 106- 1765156- NANO-MUP- 16 µg/mL- 24H.2 | 16 | 8 | >512 | Confirmed, increase in MUP MIC |
| 106- 1765156- NANO-MUP- 32 µg/mL- 24H.1 | 2 | 0.5 | >512 | Not confirmed |
| 106- 1765156- MUP- 64 µg/mL- 24H.1 | 2 | 0.5 | >512 | Not confirmed |

NANO-MUP: Nano-mupirocin; MUP: Mupirocin; RIF: Rifampicin.

**Table S9.** MICs of Nano-mupirocin, mupirocin and tetracycline for three *N. gonorrhoea* clinical isolates during multi-step passaging study.

| | | | MIC (µg/mL) at day : | | | | | | | | | | | | | | |
|---|---|---|---|---|---|---|---|---|---|---|---|---|---|---|---|---|---|
| **IHMA No** | **Organism** | **Selective agent** | **1** | **2** | **3** | **4** | **5** | **6** | **7** | **8** | **9** | **10** | **11** | **12** | **13** | **14** | **15** |
| 1073248 | *N. gonorrhoea* | **Nano-mupirocin** | 0.03 | 0.06 | 0.03 | 0.03 | 0.03 | 0.03 | 0.06 | 0.06 | 0.06 | 0.06 | 0.06 | 0.12 | 0.12 | 0.12 | 0.25 |
| | | **Mupirocin** | 0.03 | 0.03 | 0.03 | 0.03 | 0.03 | 0.015 | 0.06 | 0.06 | 0.06 | 0.12 | 0.12 | 0.12 | 0.06 | 0.12 | 0.12 |
| | | **Tetracycline** | 0.5 | 0.5 | 0.5 | 0.5 | 0.5 | 0.5 | 0.5 | 0.5 | 0.5 | 0.5 | 1 | 0.5 | 0.5 | 0.5 | 0.5 |
| 1075014 | *N. gonorrhoea* | **Nano-mupirocin** | 0.03 | 0.03 | 0.03 | 0.03 | 0.03 | 0.015 | 0.015 | 0.06 | 0.015 | 0.03 | 0.06 | 0.06 | 0.06 | 0.12 | 0.12 |
| | | **Mupirocin** | 0.015 | 0.015 | 0.03 | 0.015 | 0.015 | 0.008 | 0.03 | 0.03 | 0.03 | 0.06 | 0.06 | 0.06 | 0.03 | 0.06 | 0.06 |
| | | **Tetracycline** | 0.5 | 0.5 | 1 | 0.25 | 0.5 | 0.5 | 0.5 | 0.5 | 0.5 | 0.5 | 1 | 0.5 | 0.5 | 0.5 | 0.5 |
| 1123849 | *N. gonorrhoea* | **Nano-mupirocin** | 0.03 | 0.06 | 0.03 | 0.06 | 0.015 | 0.03 | 0.06 | 0.06 | 0.06 | 0.06 | 0.06 | 0.12 | 0.06 | 0.25 | 0.12 |
| | | **Mupirocin** | 0.015 | 0.03 | 0.03 | 0.03 | 0.008 | 0.008 | 0.03 | 0.03 | 0.03 | 0.06 | 0.06 | 0.12 | 0.06 | 0.12 | 0.12 |
| | | **Tetracycline** | 1 | 1 | 2 | 0.5 | 1 | 0.5 | 1 | 2 | 1 | 2 | 2 | 2 | 2 | 2 | 2 |

**Table S10.** MIC data for potential mutants of *N. gonorrhoea* as re-tested after five days subculture on drug-free agar

| IHMA No | Organism | Selective agent | Day | Drug | Original MIC (µg/mL) | MIC when potential mutant was isolated (µg/mL) | MIC after 5 non-selective subcultures (µg/mL) | Comment |
|---|---|---|---|---|---|---|---|---|
| 1073248 | *N. gonorrhoea* | Mupirocin | 10 | Nano-mupirocin | 0.03 | - | 0.03 | No increase of nano-mupirocin MIC observed |
| | | | | Mupirocin | 0.03 | 0.12 | 0.03 | Mutant not confirmed |
| | | | | Tetracycline | 0.5 | - | 0.5 | No increase of tetracycline MIC observed |
| | | Nano-mupirocin | 12 | Nano-mupirocin | 0.03 | 0.12 | 0.06 | Mutant not confirmed |
| | | | | Mupirocin | 0.03 | - | 0.03 | No increase of mupirocin MIC observed |
| | | | | Tetracycline | 0.5 | - | 0.5 | No increase of tetracycline MIC observed |
| | | Nano-mupirocin | 15 | Nano-mupirocin | 0.03 | 0.25 | 0.12 | Mutant confirmed for nano-mupirocin |
| | | | | Mupirocin | 0.03 | - | 0.06 | Small increase of mupirocin MIC |
| | | | | Tetracycline | 0.5 | - | 0.5 | No increase of tetracycline MIC observed |
| 1075014 | *N. gonorrhoea* | Mupirocin | 10 | Nano-mupirocin | 0.03 | - | 0.015 | No increase of nano-mupirocin MIC observed |
| | | | | Mupirocin | 0.015 | 0.06 | 0.015 | Mutant not confirmed |
| | | | | Tetracycline | 0.5 | - | 1 | No increase of tetracycline MIC observed |
| | | Nano-mupirocin | 14 | Nano-mupirocin | 0.03 | 0.12 | 0.015 | Mutant not confirmed |
| | | | | Mupirocin | 0.015 | - | 0.008 | No increase of mupirocin MIC observed |
| | | | | Tetracycline | 0.5 | - | 0.5 | No increase of tetracycline MIC observed |
| 1123849 | *N. gonorrhoea* | Mupirocin | 10 | Nano-mupirocin | 0.03 | - | 0.03 | No increase of nano-mupirocin MIC observed |
| | | | | Mupirocin | 0.015 | 0.06 | 0.015 | Mutant not confirmed |
| | | | | Tetracycline | 1 | - | 2 | No increase of tetracycline MIC observed |
| | | Nano-mupirocin | 12 | Nano-mupirocin | 0.03 | 0.12 | 0.03 | Mutant not confirmed for nano-mupirocin |
| | | | | Mupirocin | 0.015 | - | 0.03 | Small increase of mupirocin MIC observed |
| | | | | Tetracycline | 1 | - | 4 | No increase of tetracycline MIC observed |
| | | Mupirocin | 15 | Nano-mupirocin | 0.03 | - | 0.06 | No increase of nano-mupirocin MIC observed |
| | | | | Mupirocin | 0.015 | 0.12 | 0.03 | Mutant not confirmed |
| | | | | Tetracycline | 1 | - | 2 | No increase of tetracycline MIC observed |

**Table S11.** Line listing of MIC data for three *N. gonorrhoea* clinical isolates tested at different inocula and for one QC strain.

| | | | MIC agar (µg/mL) | | | |
|---|---|---|---|---|---|---|
| **Organism No** | **Organism** | **Target inoculum size (CFU/spot)** | **Nano-mupirocin diffusion** | **Mupiricin** | **Tetracycline** | **Comment** |
| ATCC 49226 | *N. gonorrhoea* | $10^4$ (Standard) | ≤0.0005 | 0.002 | 0.5 | Test performed with supplement B |
| ATCC 49226 | *N. gonorrhoea* | $10^4$ (Standard) | ≤0.0005 | 0.002 | 0.5 | Test performed with supplement B |
| ATCC 49226 | *N. gonorrhoea* | $10^4$ (Standard) | ≤0.0005 | 0.001 | 0.5 | Test performed with supplement B |
| 1073248 | *N. gonorrhoea* | $10^4$ (Standard) | 0.03 | 0.03 | 0.5 | Test performed with supplement B |
| 1073248 | *N. gonorrhoea* | $10^7$ | 0.06 | 0.06 | 1 | Test performed with supplement B |
| 1073248 | *N. gonorrhoea* | $10^6$ | 0.03 | 0.06 | 0.5 | Test performed with supplement B |
| 1073248 | *N. gonorrhoea* | $10^5$ | 0.03 | 0.03 | 0.5 | Test performed with supplement B |
| 1075014 | *N. gonorrhoea* | $10^4$ (Standard) | 0.015 | 0.03 | 0.5 | Test performed with supplement B |
| 1075014 | *N. gonorrhoea* | $10^7$ | 0.06 | 0.06 | 1 | Test performed with supplement B |
| 1075014 | *N. gonorrhoea* | $10^6$ | 0.03 | 0.06 | 1 | Test performed with supplement B |
| 1075014 | *N. gonorrhoea* | $10^5$ | 0.015 | 0.015 | 0.5 | Test performed with supplement B |
| 1123849 | *N. gonorrhoea* | $10^4$ (Standard) | 0.03 | 0.03 | 2 | Test performed with supplement B |
| 1123849 | *N. gonorrhoea* | $10^7$ | 0.06 | 0.06 | 4 | Test performed with supplement B |
| 1123849 | *N. gonorrhoea* | $10^6$ | 0.03 | 0.06 | 4 | Test performed with supplement B |
| 1123849 | *N. gonorrhoea* | $10^5$ | 0.03 | 0.03 | 2 | Test performed with supplement B |
| ATCC 49226 | *N. gonorrhoea* | $10^4$ (Standard) | ≤0.0005 | ≤0.0005 | 0.5 | Test performed with supplement VX |
| ATCC 49226 | *N. gonorrhoea* | $10^4$ (Standard) | ≤0.0005 | ≤0.0005 | 0.5 | Test performed with supplement VX |
| ATCC 49226 | *N. gonorrhoea* | $10^4$ (Standard) | ≤0.0005 | ≤0.0005 | 0.5 | Test performed with supplement VX |
| 1073248 | *N. gonorrhoea* | $10^4$ (Standard) | 0.03 | 0.015 | 0.5 | Test performed with supplement VX |
| 1073248 | *N. gonorrhoea* | $10^7$ | **0.03** | **0.03** | **4** | Test performed with supplement VX |
| 1075014 | *N. gonorrhoea* | $10^4$ (Standard) | 0.015 | 0.015 | 1 | Test performed with supplement VX |
| 1075014 | *N. gonorrhoea* | $10^7$ | **0.03** | **0.03** | **2** | Test performed with supplement VX |
| 1123849 | *N. gonorrhoea* | $10^4$ (Standard) | 0.03 | 0.015 | 2 | Test performed with supplement VX |
| 1123849 | *N. gonorrhoea* | $10^7$ | **0.03** | **0.03** | **4** | Test performed with supplement VX |

Supplement B: Becton Dickinson; Product code: 227610; Batch: 6201840; Expiry date: 07.07.2018
Supplement VX: Becton Dickinson; Product code: 233541; Batch: 7082782; Expiry date: 19.01.2020

**Table S12**. Line listing of spontaneous mutation frequency (SMF) data for *N. gonorrhoea*

| IHMA No | Organism | Inoculum | Selective agent [µg/mL] | Selective agent (× MIC) | Potential mutant at 24 h incubation | Mutants confirmed at 24 h | SMF at 24h |
|---|---|---|---|---|---|---|---|
| 1073248 | *N. gonorrhoea* | 2.12E+08 | Nano-mupirocin [0.12] | 4 × | 0 | NA | <4.72E-09 |
| 1073248 | *N. gonorrhoea* | 2.12E+08 | Nano-mupirocin [0.25] | 8 × | 0 | NA | <4.72E-09 |
| 1073248 | *N. gonorrhoea* | 2.12E+08 | Nano-mupirocin [0.5] | 16 × | 0 | NA | <4.72E-09 |
| 1073248 | *N. gonorrhoea* | 2.12E+08 | Mupirocin [0.12] | 4 × | 0 | NA | <4.72E-09 |
| 1073248 | *N. gonorrhoea* | 2.12E+08 | Mupirocin [0.25] | 8 × | 0 | NA | <4.72E-09 |
| 1073248 | *N. gonorrhoea* | 2.12E+08 | Mupirocin [0.5] | 16 × | 0 | NA | <4.72E-09 |
| 1073248 | *N. gonorrhoea* | 2.12E+08 | Tetracycline [16] | 4 × | 0 | NA | <4.72E-09 |
| 1073248 | *N. gonorrhoea* | 2.12E+08 | Tetracycline [32] | 8 × | 0 | NA | <4.72E-09 |
| 1073248 | *N. gonorrhoea* | 2.12E+08 | Tetracycline [64] | 16 × | 0 | NA | <4.72E-09 |
| 1075014 | *N. gonorrhoea* | 1.94E+08 | Nano-mupirocin [0.12] | 4 × | 0 | NA | <5.15E-09 |
| 1075014 | *N. gonorrhoea* | 1.94E+08 | Nano-mupirocin [0.25] | 8 × | 0 | NA | <5.15E-09 |
| 1075014 | *N. gonorrhoea* | 1.94E+08 | Nano-mupirocin [0.5] | 16 × | 0 | NA | <5.15E-09 |
| 1075014 | *N. gonorrhoea* | 1.94E+08 | Mupirocin [0.12] | 4 × | 0 | NA | <5.15E-09 |
| 1075014 | *N. gonorrhoea* | 1.94E+08 | Mupirocin [0.25] | 8 × | 0 | NA | <5.15E-09 |
| 1075014 | *N. gonorrhoea* | 1.94E+08 | Mupirocin [0.5] | 16 × | 0 | NA | <5.15E-09 |
| 1075014 | *N. gonorrhoea* | 1.94E+08 | Tetracycline [8] | 4 × | 0 | NA | <5.15E-09 |
| 1075014 | *N. gonorrhoea* | 1.94E+08 | Tetracycline [16] | 8 × | 0 | NA | <5.15E-09 |
| 1075014 | *N. gonorrhoea* | 1.94E+08 | Tetracycline [32] | 16 × | 0 | NA | <5.15E-09 |
| 1123849 | *N. gonorrhoea* | 9.40E+08 | Nano-mupirocin [0.12] | 4 × | 0 | NA | <1.06E-09 |
| 1123849 | *N. gonorrhoea* | 9.40E+08 | Nano-mupirocin [0.25] | 8 × | 0 | NA | <1.06E-09 |
| 1123849 | *N. gonorrhoea* | 9.40E+08 | Nano-mupirocin [0.5] | 16 × | 0 | NA | <1.06E-09 |
| 1123849 | *N. gonorrhoea* | 9.40E+08 | Mupirocin [0.12] | 4 × | 0 | NA | <1.06E-09 |
| 1123849 | *N. gonorrhoea* | 9.40E+08 | Mupirocin [0.25] | 8 × | 0 | NA | <1.06E-09 |
| 1123849 | *N. gonorrhoea* | 9.40E+08 | Mupirocin [0.5] | 16 × | 0 | NA | <1.06E-09 |
| 1123849 | *N. gonorrhoea* | 9.40E+08 | Tetracycline [16] | 4 × | 0 | NA | <1.06E-09 |
| 1123849 | *N. gonorrhoea* | 9.40E+08 | Tetracycline [32] | 8 × | 0 | NA | <1.06E-09 |
| 1123849 | *N. gonorrhoea* | 9.40E+08 | Tetracycline [64] | 16 × | 0 | NA | <1.06E-09 |

**Table S13.** Exposure parameters ($C_{max}$ and $AUC_{INF}$) for Nano-mupirocin in rates, according to dose and as compared with mean values

| | $C_{max}$/Dose (µg/mL)/(mg/kg | $AUC_{INF}$/Dose (hr*µg/mL)/(mg/kg) | % of average $C_{max}$/Dose | % of average $AUC_{INF}$/Dose |
|---|---|---|---|---|
| **Day 1** | | | | |
| **Male** | | | | |
| 10 mg/kg | 16.1 | 82.0 | 87 | 88 |
| 30 mg/kg | 18.4 | 91.5 | 100 | 98 |
| 100 mg/kg | 20.9 | 106.0 | 113 | 114 |
| Mean | 18.4 | 93.2 | | |
| **Female** | | | | |
| 10 mg/kg | 21.6 | 80.8 | 100 | 85 |
| 30 mg/kg | 19.4 | 97.2 | 90 | 102 |
| 100 mg/kg | 24.0 | 108.5 | 111 | 114 |
| Mean | 21.7 | 95.5 | | |
| **Day 14** | | | | |
| **Male** | | | | |
| 10 mg/kg | 24.8 | 123.4 | 103 | 94 |
| 30 mg/kg | 24.9 | 128.8 | 104 | 98 |
| 100 mg/kg | 22.3 | 142.1 | 93 | 108 |
| Mean | 24.0 | 131.4 | | |
| **Female** | | | | |
| 10 mg/kg | 26.3 | 105.3 | 113 | 95 |
| 30 mg/kg | 21.2 | 104.8 | 91 | 94 |
| 100 mg/kg | 22.3 | 122.7 | 96 | 111 |
| Mean | 23.3 | 110.9 | | |

## 1. Methods

*Nano-mupirocin preparation*

Nano-mupirocin was prepared as described elsewhere [1]. Briefly, the 3 lipids that form the liposomes (HSPC: cholesterol: mPEG DSPE(2000 Da)), were dissolved in ethanol at a weight ratio of 3:1:1, and were mechanically hydrated by stirring at 65 °C with 200 mM calcium acetate pH 5.5 solution containing 15% (*w/w*) HPCD. The liposomal dispersions were downsized by stepwise extrusion using polycarbonate filter membranes of defined pore size and subjected to diafiltration using a Quixstand Benchtop System (GE Healthcare, Uppsala, Sweden) against isotonic 10% sucrose. These 'nano-liposomes.' exhibiting a trans membrane acetate gradient and high intraliposomal concentration of calcium ions and of HPCD were remotely loaded by 10 min incubation at 65 °C with a 15 mg/mL mupirocin solution in 200 mM phosphate buffer, pH 6.3.

The fluorescently labeled lissamine-rhodamine B phosphatidylethanolamine (LRPE)-Nano-mupirocin liposomes (LRPE-Nano-mupirocin) were prepared by incubating Nano-mupirocin with

1,2-dioleoyl-sn-glycero-3-phosphoethanolamine-N-(lissamine rhodamine B sulfonyl) (LR-PE) (Avanti Polar Lipids, AL, USA) dissolved in ethanol at a final molar ratio of 0.2 mole % of the liposomal lipids for 5 min at 37 °C and then overnight at 4 °C. Note that LRPE is labeled in the polar head group of the phospholipid and therefore is retained within the nanoliposomes.

Nanoliposomes size was measured by the dynamic light scattering method, performed with a Zetasizer Nano Series ZEN3600F instrument (Malvern Instruments, Malvern, UK).

## 2. Bioanalytical methods

Bioanalytical testing was performed at two sites for the PK and PK/BD studies. Slightly different assays were used at these, as described below.

### *2.1. Pharmacokinetic study*

Sample preparation

Plasma samples (20 μL) were extracted in 1 mL of 0.1% formic acid in acetonitrile containing 100 ng/mL mycophenolic acid (internal standard, IS). Following vortexing and centrifugation, the upper phase was diluted × 5 in water. Mupirocin stock standards were prepared in methanol. Further dilutions of the standards were prepared in a 50/50 acetonitrile/water solution.

LC-MS/MS method

A HPLC-tandem mass spectrometry (LC-MS/MS) assay was used to quantify mupirocin in the plasma samples, with acceptable accuracy and precision, over the concentration range of 0.500–500 μg/mL. Chromatography was performed under reverse phase conditions using a Shimadzu (Kyoto, Japan) HPLC System. The analytical column used was Synergi, Polar-RP, 2.5 μm, 100A, 50 × 2.0 mm (Phenomenex, Torrance, CA, USA). The injection volume was 5 μL, the oven temperature was maintained at 40 °C and the autosampler tray temperature was maintained at 5 °C. A mixture of water:MeOH:formic acid (80:20:1) was used for washing the needle prior to each injection cycle.

The chromatographic separation was achieved using a linear gradient program at a constant flow rate of 0.5 mL/min over a total run time of 4 min. An outline of the mobile phase gradient program is summarized as below:

| Time (min) | Solvent A (%) 0.1% Formic Acid in Water | Solvent B (%) 0.1% Formic Acid in Acetonitrile |
|---|---|---|
| 0.0 | 90 | 10 |
| 1.0 | 80 | 20 |
| 2.5 | 20 | 80 |
| 3.0 | 20 | 80 |
| 3.1 | 90 | 10 |
| 4.0 | 90 | 10 |

Mupirocin and mycophenolic acid (IS) were detected in positive ion mode using an AB Sciex API 4000 mass spectrometer (Framingham, MA, USA) with electrospray ionization (ESI) employing the 'multiple reaction monitoring' (MRM) mode of acquisition. The TurboIonspray® probe temperature was set at 500 °C with the ion spray voltage at 2500 V. The curtain gas was set at 30 psi. The nebulizer gas (Gas 1) was set to 50 psi, the turbo heater gas (Gas 2) at 50 psi and the collision gas (CAD) at 8 psi. The entrance potentials (EP), collision energy potentials (CE), collision cell exit potentials (CXP) and declustering potentials (DP) for the monitored transitions are given below. The dwell time was 100 ms. Data acquisition and analysis were performed with Analyst 1.6.2 software distributed by AB Sciex.

Multiple reaction monitoring (MRM) transitions and parameters for Mupirocin and the IS in positive ion mode

| | Precursor (m/z) | Product (m/z) | DP (V) | EP (V) | CE (eV) | CXP (V) | Rt (min) |
|---|---|---|---|---|---|---|---|
| Mupirocin | 501.5 | 309.2 | 63.0 | 6.20 | 21.0 | 7.9 | 2.71 |
| IS | 321.3 | 207.2 | 61.9 | 9.24 | 31.4 | 12.0 | 2.83 |

m/z: mass to charge ratio; DP: declustering potential; EP: entrance potential; CE: collision energy; CXP: collision cell exit potential; V: volts; eV: electronvolts; Rt: retention time. .

*2.2. In-house PK and BD studies*

Preparation of plasma samples

Plasma samples were spiked with mycophenolic acid (IS) to a concentration of 10 μg/mL and diluted either 5-fold or 50-fold with acetonitrile. Samples were then vortexed, centrifuged, and the upper phase was diluted 1:1 with double distilled water. Samples were analyzed by the LCMS/MS method. Mupirocin concentrations were calculated based on a calibration curve of mupirocin in plasma from untreated mice.

Preparation of swab samples

Following sampling, swabs were weighed and immediately placed in a 15 mL tube containing 2 mL of acetonitrile. To each tube, 25 μL of 3 μg/mL mycophenolic acid (IS) in methanol were added. The tubes were vortexed vigorously for 1 min, then the swab was removed and the tube was placed in an evaporator until complete dryness was achieved. Next, 75 μL of MeOH was added followed by vortex (1 min), sonication (0.5 min), and centrifugation (10 min, 4,000 rpm). The upper phase was collected and analyzed by the LCMS/MS method. The mupirocin concentration in the swab was calculated based on a calibration curve of mupirocin spiked into swabs obtained from untreated mice. The absolute amount of mupirocin recovered from each swab was related to the average weight of vaginal secretion 3.45 mg (SD = 2.53) collected per swab, to calculate the local mupirocin concentration as μg/g. This average was based on 18 measurements, including swabs from both treated and untreated mice.

LC-MS/MS method

Chromatography was performed under reverse phase conditions using a Thermo Scientific system (San Jose, CA, USA), which includes a Dionex pump with degasser module and an Accela Autosampler. The chromatographic separations were performed on a Kinetex™ (Phenomenex, Torrance, CA, USA) column (C18, 2.6 μm particle size, 100 Å pore size, 50 × 2.1 mm), protected by a SecurityGuard™ (Phenomenex, Torrance, CA, USA) cartridge (C18, 4 × 2.1 mm). The injection volume was 5 μL, the oven temperature was maintained at 40 °C, and the autosampler tray temperature at 4 °C. The chromatographic separation was achieved using a linear gradient program at a constant flow rate of 0.35 mL/min over a total run time of 8 min. An outline of the mobile phase gradient program is summarized in the table below.

| Time (min) | Solvent A (%) 0.1% formic acid in water | Solvent B (%) 0.1% formic acid in acetonitrile |
|---|---|---|
| 0.0 | 90 | 10 |
| 2.3 | 55 | 45 |
| 4.0 | 55 | 45 |
| 4.3 | 10 | 90 |
| 5.0 | 10 | 90 |
| 5.5 | 90 | 10 |
| 8.0 | 90 | 10 |

The column effluent was diverted away from the mass spectrometer during the first 1.3 min of the run. A mixture of water:MeOH (1:1) was used for washing the needle prior to each injection cycle. Mupirocin and mycophenolic acid (IS) were detected by a TSQ Quantum Access Max mass spectrometer in positive ion mode using electron spray ionization (ESI) and the multiple reaction monitoring (MRM) mode of acquisition. The spray voltage, sheath and auxiliary gas were set at 4500 V, 30 and 35 (arbitrary units), respectively. The capillary transfer tube temperature was set at 250 °C and the tube lens was set at 85 V for mupirocin and 88V for mycophenolic acid. The vaporizer temperature within the H-ESI source was 400 °C. The scan time was 40 ms.

The following transitions were monitored:

Mupirocin: m/z 501 → 309 (quantifier), collision energy (CE) 15 V and m/z 501 → 327 (qualifier), CE 12 V, retention time (RT) 4.1 min.

Mycophenolic acid: m/z 321 → 207 (quantifier), CE 21 V and m/z 321 → 303 (qualifier), CE 10 V, retention time (RT) 4.0 min.

Data acquisition and processing were carried out using the Xcalibur program (Thermo Scientific, San Jose, CA, USA). Quantitative calibration (1–5000 ng/mL) was performed before every batch of samples. The calibration curves were created using peak-area ratios (analyte versus internal standard). The calibration curve ($y = a + bx$) was obtained by weighted (1/y) linear least-squares regression of the measured peak-area ratios ($y$) of mupirocin/mycophenolic acid versus the concentration of mupirocin added to the plasma ($x$). The limit of quantification (LOQ) was 1 ng/mL for mupirocin.
